# Perspectives of people with dementia and carers on advance care planning and end-of-life care: A systematic review and thematic synthesis of qualitative studies

**DOI:** 10.1177/0269216318809571

**Published:** 2018-11-08

**Authors:** Marcus Sellars, Olivia Chung, Linda Nolte, Allison Tong, Dimity Pond, Deirdre Fetherstonhaugh, Fran McInerney, Craig Sinclair, Karen M Detering

**Affiliations:** 1 Advance Care Planning Australia, Austin Health, Melbourne, VIC, Australia; 2 Sydney Medical School, The University of Sydney, Sydney, NSW, Australia; 3 Sydney School of Public Health, The University of Sydney, Sydney, NSW, Australia; 4 School of Medicine and Public Health (General Practice), The University of Newcastle, Callaghan, NSW, Australia; 5 Australian Centre for Evidence Based Aged Care, La Trobe University, Melbourne, VIC, Australia; 6 Wicking Dementia Research and Education Centre, College of Health and Medicine, University of Tasmania, Hobart, TAS, Australia; 7 Rural Clinical School of Western Australia, University of Western Australia, Albany, WA, Australia; 8 Faculty of Medicine, Dentistry and Health Science, University of Melbourne, Melbourne, VIC, Australia

**Keywords:** Dementia, advance care planning, carers, qualitative research, end-of-life, systematic review

## Abstract

**Background:**

Advance care planning aims to ensure that care received during serious and chronic illness is consistent with the person’s values, preferences and goals. However, less than 40% of people with dementia undertake advance care planning internationally.

**Aim:**

This study aims to describe the perspectives of people with dementia and their carers on advance care planning and end-of-life care.

**Design:**

Systematic review and thematic synthesis of qualitative studies.

**Data sources:**

Electronic databases were searched from inception to July 2018.

**Results:**

From 84 studies involving 389 people with dementia and 1864 carers, five themes were identified: avoiding dehumanising treatment and care (remaining connected, delaying institutionalisation, rejecting the burdens of futile treatment); confronting emotionally difficult conversations (signifying death, unpreparedness to face impending cognitive decline, locked into a pathway); navigating existential tensions (accepting inevitable incapacity and death, fear of being responsible for cause of death, alleviating decisional responsibility); defining personal autonomy (struggling with unknown preferences, depending on carer advocacy, justifying treatments for health deteriorations); and lacking confidence in healthcare settings (distrusting clinicians’ mastery and knowledge, making uninformed choices, deprived of hospice access and support at end of life).

**Conclusion:**

People with dementia and their carers felt uncertain in making treatment decisions in the context of advance care planning and end-of-life care. Advance care planning strategies that attend to people’s uncertainty in decision-making may help to empower people with dementia and carers and strengthen person-centred care in this context.

**What is already known about the topic?**

- Advance care planning (ACP) supports people to consider and communicate their current and future treatment goals. However, only up to 40% of people with dementia undertake ACP worldwide.
- People with dementia receive sub-optimal care at end of life, including overly aggressive treatments, low rates of palliative care referrals and poor pain and symptom management.

**What this paper adds?**

- People with dementia and their carers felt uncertain in making decisions in the context of ACP and end-of-life care.
- People with dementia and their carers had to confront emotionally difficult conversations and navigate existential tensions during ACP; while also feeling a sense of distrust and a lack of confidence in the information and support available to them in healthcare settings.
- Carers needed to overcome uncertainty if the person with dementia had not previously expressed their preferences; they felt adhering to the ACP preferences of the person with dementia would make them responsible for the person’s death; or they experienced disagreement with clinicians when advocating for the preferences of the person with dementia.

**Implications for practice, theory or policy**

- Health professionals who are involved in ACP and end-of-life care in dementia should demonstrate empathy and aim to facilitate acceptance of the inevitable cognitive decline and death in dementia and provide an understanding of the decisions that may need to be made along the trajectory of dementia.
- Future ACP strategies should attend to potential uncertainties that may arise when carers are attempting to adhere to the person with dementia’s ACP preferences at end of life.

## Introduction

The increasing prevalence of dementia is an international public health priority, affecting an estimated 47 million people globally, and is expected to nearly triple in prevalence by 2050.^1^ Dementia is a progressive and terminal illness, characterised by impaired memory, thinking, reasoning and communication. For people with dementia, the ability to make decisions, plan for the future and perform daily self-care ultimately deteriorates as the disease progresses.^2^ Because of this, caregiving for people with dementia can be emotionally challenging, particularly when facing decisions about the person’s future medical care,^3,4^ such as whether to consent to life-sustaining treatments.^5^

Advance care planning (ACP) supports people to consider and communicate their future treatment preferences in the context of their own goals and values. It is an ongoing process in which a person may need to appoint a substitute decision-maker and document their preferences for care in an advance care directive or advance care plan.^6^ The goal of ACP is to ensure that people receive treatment and care consistent with their goals, values and preferences during serious and chronic illness.^7^ Yet ACP is estimated to occur with only 3%–39%^8–10^ of people with dementia internationally. People with dementia receive sub-optimal care at end of life,^11^ including overly aggressive treatments, low palliative care referrals^12^ and poor pain and symptom management.^13^ Moreover, although people with dementia and their carers believe ACP is relevant to people with dementia and it should be completed early in the illness trajectory,^14,15^ they may not feel comfortable discussing ACP because of fear of future cognitive decline.^16,17^

Qualitative research methods are used to elicit the attitudes and beliefs of participants to generate in-depth and nuanced insight into their perspectives.^18,19^ A systematic review and synthesis of qualitative studies can bring together data across different populations and contexts, beyond a single primary study. This allows a more comprehensive understanding to inform clinical practice regarding ACP and end-of-life care in dementia that accords with their values and preferences.^20,21^ This study aims to describe the perspectives of people with dementia and their carers concerning ACP and end-of-life care in dementia, which may inform strategies that will maximise quality of care and quality of life outcomes in this vulnerable population.

## Methods

We followed the Enhancing Transparency of Reporting the Synthesis of Qualitative Research (ENTREQ) framework^21^ and used thematic synthesis as described by Thomas and Harden.^20^ Thematic synthesis is used to formalise the identification and development of themes from multiple primary studies and subsequently enabled the development of a comprehensive conceptual framework for this study that can explain the experiences and perceptions of people with dementia and their carers.

### Data sources and searches

The search strategies are provided in Supplementary Table S1. We searched MEDLINE, Embase, PsycINFO and CINAHL from database inception to 6 July 2018. Google Scholar, PubMed and reference lists of relevant articles were also searched. Two reviewers (M.S. and O.C.) independently screened the search results, initially by title and abstract, then the full texts of potentially relevant studies for eligibility. Studies that did not meet the inclusion criteria were excluded.

### Selection criteria

Qualitative studies were eligible if they reported the experiences of people with dementia and carers and perspectives of ACP and end-of-life care in dementia. Study participants had to include adults aged 18 years or older diagnosed with dementia of any type or stage in the illness trajectory, and/or carers (i.e. family member, friend or other appointed substitute decision-maker) who provided unpaid care and support to a person with dementia. Studies across all care settings were eligible for inclusion. ACP was defined as any intervention aimed at supporting people to consider and communicate their current and future treatment goals in the context of their own preferences and values. End-of-life care was defined as any treatment or care around death or the dying process. Studies involving mixed methods (including surveys) or process evaluation that reported qualitative data were included if qualitative data could be extracted. Studies were excluded if they exclusively examined euthanasia or ‘assisted suicide’, or reported only quantitative data. We also excluded non-English articles to minimise misinterpretation of any linguistic and cultural nuances.

### Quality assessment

We assessed each primary study for comprehensiveness of reporting, which can provide details for readers to assess the trustworthiness and transferability of study findings. We used an adapted consolidated criteria for reporting qualitative health research (COREQ)^22^ framework, which included criteria specific to the research team, study methods, study setting, analysis and interpretations. Two reviewers (M.S. and O.C.) independently assessed each study and resolved discrepancies through discussion.

### Data analysis

Consistent with thematic synthesis guidelines,^20^ participant quotations and text under the results/findings or conclusion/discussion sections were imported for each article into HyperRESEARCH (ResearchWare Inc. version 3.7.5; 2015) software. One investigator (M.S.) performed line-by-line coding of the findings from studies generated by the database search, conceptualised the data and inductively identified concepts. Text was then coded into existing concepts or a new concept was created as required (M.S. and O.C). Similar concepts were grouped into themes and subthemes. Conceptual links among themes were identified (M.S. and O.C.) to extend the findings offered by the primary studies and develop an analytical thematic schema. To ensure that coding captured all relevant issues and reflected the primary data, researcher triangulation was used, in which two reviewers (M.S. and O.C.) independently reviewed the preliminary themes and analytical framework, and discussed the addition or revision of themes with all the authors.

## Results

### Literature search

**Figure 1.**
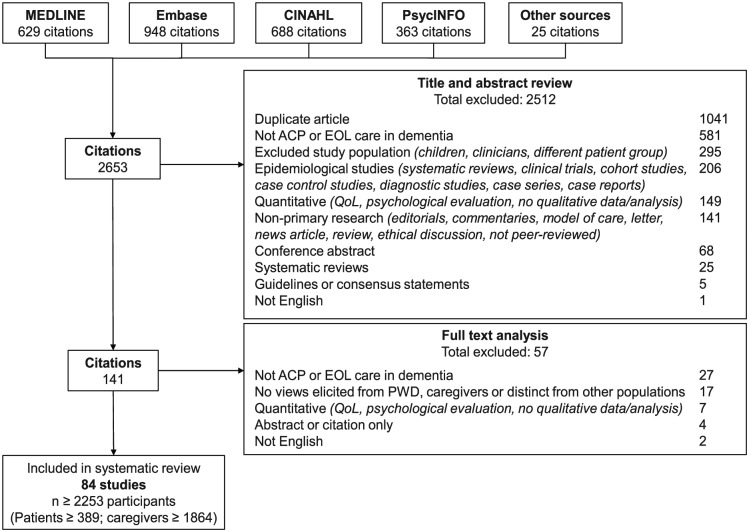
Search results. ACP: advance care planning; EOL: end-of-life; QoL: quality of life.

From 2653 articles identified in the search, we included 81 articles involving at least 389 people with dementia and 1864 carers from 14 countries (Figure 1). Two studies did not report the number of participants. The age of people with dementia ranged from 46 to 95 years, while carers’ ages ranged from 18 to 95 years. The characteristics of the included studies are summarised in Table 1, with the details of each study provided in Supplementary Materials (Supplementary Table S2). The included studies were published from 1996 to 2018. Fifty (60%) studies reported the stage of dementia, with advanced stage being most commonly reported (75%). Of the 76 studies involving carers, 62 reported on the carer’s relationship to the person with dementia, which included spouse/partner (56 studies), child (57 studies), grandchild (9 studies), sibling (13 studies) and other (29 studies).

**Table 1.** Characteristics of included studies (*N* = 84).

Characteristics	Number of studies (%)
Country	
United States	27 (32)
United Kingdom	22 (26)
Europe	18 (21)
Australia	9 (11)
Canada	6 (7)
Asia	2 (2)
Study population	
People with dementia	7 (9)
Carer	59 (74)
Person with dementia and carer	15 (17)
Care setting (*N* = 77)^a^	
Care home (e.g. nursing home, residential aged care facility)	62 (81)
Community (e.g. home, assisted living)	37 (48)
Hospital	30 (39)
Hospice	12 (16)
Stage of dementia (*N* = 42)^b^	
Mild/early stage	14 (32)
Moderate/middle stage	6 (14)
Advanced	33 (75)
Data collection method^c^	
Interview	70 (83)
Focus group	10 (12)
Other (e.g. observation, nominal group technique, Q-methodology)	10 (12)

a 32 studies reported multiple care settings.

b 6 studies reported multiple dementia stages.

c 5 studies reported multiple data collection methods.

### Comprehensiveness of reporting

The comprehensiveness of reporting was variable, with studies reporting 2 to 22 of the 34 items included in the framework for assessing the reporting of qualitative studies (Table 2). The sampling strategy was described in 53 (65%) studies. Theoretical or data saturation,^19^ whereby subsequent data collection identified few or no novel concepts, was reported in 18 (22%) studies. Member checking, whereby participant feedback is obtained for preliminary findings, was reported in five (6%) studies, while the number of data coders (e.g. investigator triangulation used in data analysis) was reported in 57 (70%) of studies.

**Table 2.** Comprehensiveness of reporting in included studies.

Item	Studies reporting each item	No. of studies (%)
Personal characteristics		
Interviewer or facilitator identified	3, 4, 15, 16, 23–56	38 (47)
Credentials	16, 24–27, 29, 30, 33–39, 42, 48, 50, 56–62, 63–72	35 (43)
Occupation	15, 16, 24, 31, 33, 35–38, 40, 42, 43, 45, 47–49, 53, 54, 56, 60, 65–67, 73–80	31 (38)
Sex	4, 5, 14, 16, 23–25, 27–30, 33, 37–40, 42, 44, 46–48, 50, 51, 53, 59, 60, 62, 63, 69–73, 75, 77, 81–89	45 (56)
Experience and training (in qualitative)	16, 23, 25, 27, 32, 38, 43, 47, 49, 50, 56, 78, 90	13 (16)
Relationship with participants		
Relationship established before study started	3, 39, 46, 53, 55, 56, 91	7 (9)
Participant knowledge of interviewer		0 (0)
Interviewer characteristics (e.g. bias)	27, 28, 37, 45, 46, 48, 49, 52	8 (10)
Theoretical framework		
Methodological theory identified	14, 27, 28, 37–39, 44, 46–50, 52, 54–56, 62, 66, 69, 82–84, 92	23 (28)
Sampling method (e.g. snowball, purposive)	3, 5, 14, 16, 17, 23–28, 31, 32, 34, 35, 39, 40–42, 44, 46–49, 54–56, 58, 59, 61, 62, 64, 67–70, 73–75, 78, 79, 81–85, 87, 88, 90, 92–97	53 (65)
Method of approach or recruitment	3–5, 14, 15, 27, 29, 30, 31, 32, 34, 35, 40–44, 48–50, 54, 55, 62–65, 68–71, 73–75, 78, 79, 81–83, 87, 88, 92, 93, 95–98	48 (59)
Sample size	3–5, 14–17, 23–99	81 (100)
Non-participation (e.g. number or reasons)	15–17, 25, 28–31, 38, 40–42, 47–50, 55, 59, 64, 65, 75–78, 82, 88, 90, 92, 94, 96, 98	31 (38)
Setting		
Setting of data collection	3, 14, 15, 17, 24, 28, 30–32, 34, 35, 37, 38, 40, 42, 43, 45, 47, 48, 49, 52, 53, 54, 55, 57, 59, 61–63, 65, 66, 69, 71–73, 76–78, 80, 82–84, 86, 90, 91, 93, 94, 96, 98	48 (59)
Presence of non-participants	45, 66, 75	4 (5)
Description of sample	3–5, 14, 16, 17, 24–27, 29–38, 40–43, 46–50, 52–57, 59, 61–67, 69, 70, 72–79, 81–85, 87–89, 91–98	69 (85)
Data collection		
Interview guide (e.g. questions, prompts)	3–5, 14–17, 23–35, 37–47, 49–59, 61–65, 67, 69–71, 73–75, 77–83, 85–90, 93–99	72 (89)
Repeat interviews	26, 36, 41, 46, 48, 49, 53, 69, 71, 85	10 (12)
Audio or visual recording	3–5, 14–17, 23–37, 39–43, 45–55, 59, 61–63, 65, 69–73, 75–85, 87, 88, 90–98	72 (89)
Field notes	3, 8, 16, 23, 37, 44, 45, 47, 49, 50, 52, 53, 56, 57, 59, 73, 75, 76, 82, 84–86, 94	22 (27)
Duration	3–5, 14–17, 23, 24, 26–29, 31–43, 49, 50, 54–56, 60–65, 69–71, 73–78, 81–84, 87, 88, 91–97	53 (65)
Data (or theoretical) saturation^a^	4, 16, 17, 28, 32, 42, 52, 54, 55, 70, 73, 79, 84, 85, 93, 95, 98	18 (22)
Transcripts returned to participants	5, 63, 70, 84	4 (5)
Data analysis		
Number of data coders	3, 4, 15–17, 23–28, 30–37, 39–43, 45–49, 52–57, 68–79, 82, 83, 85, 87, 91, 93–95, 98	57 (70)
Description of coding tree	3–5, 14, 15, 17, 24–27, 30–35, 38, 43, 46, 47, 49, 54, 56, 58, 59, 63, 65, 68, 70, 72–74, 76–79, 81, 84, 85, 87, 90, 91, 93–96	45 (56)
Derivation of themes (e.g. inductive)	14–17, 23–36, 40–51, 53–59, 61–63, 65, 66, 68–73, 75–79, 81–85, 87, 90–98	68 (84)
Use of software	4, 5, 15–17, 23–26, 29, 31, 33–35, 38, 41–43, 46, 49, 50, 55, 61, 63, 66, 70, 75, 78, 79, 82, 85, 95, 96	33 (41)
Participant’s feedback or member checking	28, 32, 44, 53, 63, 85	5 (6)
Reporting		
Participant quotations provided	3–5, 14–17, 23–28, 30–59, 61–73, 75–98	80 (99)
Data and findings consistent	3–5, 14–17, 23–28, 30–36, 38–40, 43, 44, 46–59, 61–94, 96–98	76 (94)
Clarity of major themes	3–5, 14–17, 23–38, 40, 44, 46–56, 58, 59, 61, 63–73, 75–85, 87–94, 96, 97	71 (88)
Clarity of minor themes	17, 25, 29–31, 44, 46, 51, 52, 58, 67, 69, 73, 76, 78, 79, 85, 87, 92, 96	20 (25)

a Data saturation is defined as when few or no new data is generated subsequent to data collection.

### Synthesis

**Figure 2.**
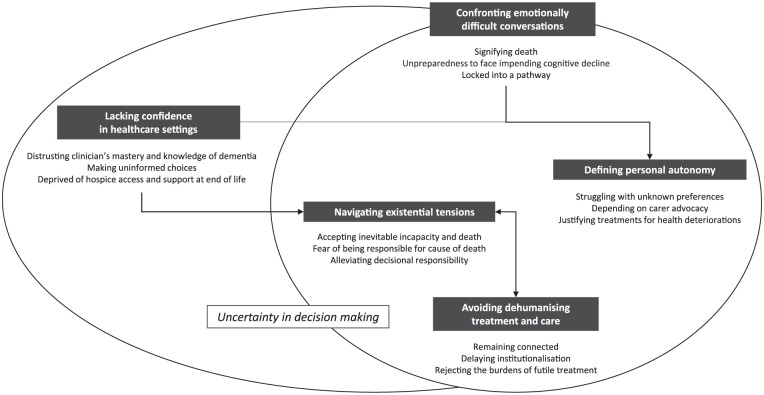
Thematic schema of people with dementia and carer’s perspectives of ACP and end-of-life care. For people with dementia and their carers, ACP and end-of-life care was characterised by a sense of uncertainty in decision-making. ACP required some to confront emotionally difficult conversations and some carers felt unprepared in the act of adhering to ACP preferences and making end-of-life decisions on behalf of the person with dementia. In addition, a lack of confidence in healthcare settings contributed to carer uncertainty while they navigated existential tensions nearing death. To overcome these challenges, people with dementia and their carers expressed needs and conditions to avoid dehumanising treatment and care.

We identified five themes: avoiding dehumanising treatment and care, confronting emotionally difficult conversations, navigating existential tensions, defining personal autonomy and lacking confidence in healthcare settings. These are detailed in the following section. The themes were relevant to both people with dementia and carers unless specified otherwise. Selected quotations to illustrate each theme are provided in Table 3. Conceptual links among themes are presented in Figure 2.

**Table 3.** Illustrative quotations by theme.

Subtheme	Representative quotations	Contributing studies^a^
Avoiding dehumanising treatment and care		
Remaining connected	‘I think to me as long as I’ve got [husband], as long as we’re together that’s all that matters to me’. (Person with dementia)^54^ ‘That’s a goal for me; to keep her active and social. She may not be chatting with anybody when we go out to dinner, but it’s part of being connected to the rest of the world’. (Carer)^87^ ‘We got (husband) out every day that the sun was shining into the grass in the garden. He’s an ornithologist, loved birds, could tell them all by their sound. And the smell of the jasmine was around and you could see him responding to it. So, in terms of those things, and just feeling the warmth of the sun, [it] was incredibly important’. (Carer)^23^ ‘She [staff carer] spent two or three hours every night reading the bible to my mother. They were looking after her, they kept making sure she was comfortable, they kept moistening her face, and going in all the time and talking to her’. (Carer)^54^	23, 39, 44, 51, 53, 54, 57, 69, 70, 73, 75, 77, 78, 86, 87, 93, 96
Delaying institutionalisation	‘Look after me with care. Don’t treat me like a vegetable, like a mad person’. (Person with dementia)^24^ ‘Keep me clean, no dirty clothes, no food on my face. Fresh underwear, oh Lord is a must. I want to look like how I always looked’. (Person with dementia)^58^ ‘It was [at] the discharge suite. I walked in to pick him up and he was sat in a green gown, half naked. His legs exposed. He was soaking wet, soiled himself and he had somebody else’s glasses on. You know he had no sheet or blanket or anything covering his dignity’. (Carer)^73^ ‘He was agitated and frustrated in hospital. He didn’t know where he was, why he was there and was aggressive a couple of times. He wandered and when (they) tried to get him back he hit out. They got security in, sedated him and he slept for 24 hours with no food or drink. That was in the last three months of his life’. (Carer)^94^ ‘I think all those little things made [him] really comfortable physically. [The caring staff] were very understanding, just down to how warm he was, how cold he was, what he needed on top of him, his pillows, just very small details like giving his face and hands a wipe because they felt a bit clammy, or changing quilts, changing sheets. It was excellent care’. (Carer)^77^	16, 24, 25, 28, 33, 37, 39, 49, 54, 56, 58, 62, 64, 66, 67, 69, 73, 77, 79, 81, 83, 84, 89, 93, 94
Rejecting the burdens of futile treatment	‘It’s about quality of life. I would hate to be kept alive beyond my natural time, especially if I couldn’t speak or lost my motions. I would rather just slip away. I would hate to be in pain and would want good pain relief’. (Person with dementia)^60^ ‘We’re talking about an irreversible disease that leads to death. It’s better to let nature take its course. Make her comfortable, with no pain, and [allow her] a dignified death. For me, using a stomach tube is futile. And so, there is no question about that, I am very comfortable with all this’. (Carer)^26^‘When it got very close to the end of his life, they did ask me whether I wanted him to be fed through his stomach. The doctor gave me the facts and didn’t try to influence me, but it seemed that to prolong his life would be cruelty’. (Carer)^99^	5, 14, 17, 26, 28, 38, 42, 59, 60, 79, 82, 83, 86
Confronting emotionally difficult conversations		
Signifying death	‘I don’t think I could write it down. I would feel I was putting him to his death’. (Carer)^4^ ‘Mother is one of [those people] that can’t talk about things like that because, as soon as you do, she feels she’s dying. [It’s] a very tender subject. How to do it, I don’t know at the moment. I just have to see how things develop’. (Carer)^5^	4, 5, 14, 26, 61
Unpreparedness to face impending cognitive decline	‘As far as I’m concerned I know I have it, but I’m not aware how it affects me. I don’t think about it and I don’t really want to know. I know it sounds a bit Irish, but if you don’t know about it won’t happen!’ (Person with dementia)^60^ ‘We didn’t ever have that discussion, ’cause we didn’t think [dementia] would happen to her, and by the time things started to happen, it was too late’. (Carer)^54^ ‘I procrastinated for a couple of years about it [advance care planning], because I just couldn’t deal with it emotionally. It was something I knew we had to do before he got too sick, so that he could sign all the papers, and I just, I couldn’t deal with it for a while. So, when I finally got it together, it was a good thing, because we were right on the verge there, of being at a point where a doctor might not have been comfortable signing the paper’. (Carer)^26^	4, 14, 17, 24, 26, 38, 42, 54, 60, 65, 88, 92, 95
Locked into a pathway	‘Well, I’ll have a plan myself … But it might not involve any medical people or care people. Formal [plan], no. But I’ll have a good idea what I want to do [and] I’ll discuss it with my wife’. (Person with dementia)^54^ ‘My slight fear about advanced care planning [is] that it [may] take away [control] from individuals, even though it’s prepared by an individual; you have to tick certain boxes’. (Carer)^24^ Now, with dementia, it’s not quite so easy, because you might want one thing earlier on and you mightn’t have the capacity to say what you want later on. (Carer)^5^	4, 5, 15, 24, 27, 29, 48, 54
Navigating existential tensions		
Accepting inevitable incapacity and death	‘I’m not afraid of these things, there’s nothing to be afraid of. Being dead doesn’t hurt, you know. Everybody dies. So there you go. That’s all’. (Person with dementia)^34^‘Accept [death] as natural as otherwise you will be unhappy. Can’t treat what you’re dying of, that’s why you’re dying’. (Person with dementia)^58^ ‘We knew with this disease that eventually he wasn’t going to be able to make decisions. Somebody had to make them for him, and it was I who he wanted to make them’. (Carer)^95^ ‘We did [advance care planning] long ago, knowing that someday we were going to reach this stage [of advanced dementia], and that while we were capable at the time, we should be the ones to make the decisions. Not wait until there was an emergency, or until both of us were so senile, that we didn’t know what in the heck was going on. So the decisions were made when he was still able’. (Carer)^26^	5, 14, 17, 26, 30, 31, 34, 38–40, 52, 55, 56, 58, 59, 62, 63, 70, 82, 86, 95–97
Fear of being responsible for cause of death	‘You feel like you’re killing her by not taking her to the hospital, because that’s just your natural reaction is you want to save somebody. And to say, “Please don’t take her to the hospital” – I mean, it’s a horrible thing to have to say about your mother! You know? And yet, it really is much more loving’. (Carer)^28^	5, 28, 31, 38, 45, 59, 61, 82,
	‘You feel like you’re killing her by not taking her to the hospital, because that’s just your natural reaction is you want to save somebody. And to say, “Please don’t take her to the hospital” – I mean, it’s a horrible thing to have to say about your mother! And yet, it really is much more loving’. (Carer)^28^	
	‘I can’t implement [the preferences of the person with dementia as articulated in the written advance directive]. I can’t do nothing because he is going to die in front of my eyes and he doesn’t have to die now … I actually felt that I’d breached his wishes’. (Carer)^5^	
Alleviating decisional responsibility	‘They’ve spelled everything outright in their health care proxy. And that is what we have used as [a] kind of bible for our decisions’. (Carer)^30^‘We had talked to her when she was in her mid-80s and very much alive and vibrant, and discussed what she wanted when this situation arose. She was very clear that when it’s time it’s time. And she was anxious to sign a DNR, [as] she didn’t want to be kept alive and wanted to die peacefully and comfortably. She was very much a part of the decision process and we just fulfilled those wishes for her’. (Carer)^30^ ‘I think it is great. It just takes burden from anybody else. That is what advance directives are supposed to do. You get to decide and not burden somebody else with that decision’. (Carer)^45^	4, 5, 14, 15, 28–30, 56, 93, 98
Defining patient autonomy		
Struggling with unknown preferences	It is difficult to understand what’s going on in her brain. The only measure I’ve got is that she is calm, contented, [but] my view [is] that she is [not] where she [wants] to be. I think one of the things to bring out is a living will, if we’d done it, because, I mean, she’s living on a knife-edge”(Carer)^56^ ‘I mean I’ve known him since I was 18, and I’m 78 now. We never ever talked about dying … now I wish we had done. It’s a funny thing, because if me and [husband] had discussed it, I would [be able to] say ’right I’m doing his wishes’, but now I don’t know’. (Carer)^54^ ‘I wonder about doing the right thing as I did not have a lot of contact with her up until she became ill. I try and think about what she would have wanted when she was younger or what I would have liked. I also think about how our parents died. I suppose you just have to do the best you can’. (Carer)’^14^	14, 26, 54, 56, 61, 91, 93
Depending on carer advocacy	‘My family knows what I told them I want … all they have to do is tell everyone else’. (Person with dementia)^58^ ‘It’s good to have the plan, but I also think you need the backup of your family to see that the plan is implemented to its best outcome’. (Person with dementia)^54^ “I think that they [doctors] try to push it, from their own perspective, like they don’t want anybody to die on their watch. That sounds awful, but I feel that way, because there was so much pressure, “she needs a feeding tube, she needs this central line, she needs all this medication.” I [thought] you can’t do this to her’. (Carer)^31^	3, 31, 40, 54, 58, 59, 69, 82, 98
Justifying treatments for health deteriorations	‘If she has pneumonia, I want it to be treated. Because, with pneumonia, people get over it without many problems afterwards. As long as it’s a disease that with modern medicine we can say is benign, or at least, not fatal, then I think that we have to treat it as long as she is no worse than she is right now’. (Carer)^82^ ‘I think I would want the feeding tube, because the rest of her body wasn’t going. The only thing that was holding her up was the fact that she wasn’t eating, [so] she’s starving herself to death rather than dying. I would ask for a feeding tube until her body seemed to be all complete, be going. I don’t want her to starve’. (Carer)^38^	5, 38, 45, 48, 52, 59, 79, 82, 88, 91
Lacking confidence in healthcare settings		
Distrusting clinician’s mastery and knowledge	‘A doctor or social worker, one will say one thing and one will say something else. One will treat it as what it is, and one will just say well it’s only memory problems. I feel like giving them a slap, and saying yes I know that’s what it is now but who’s to say what it’s going to be in two years, five years, or ten years’. (Person with dementia)^4^ ‘When you ask a doctor or a nurse how long, what is the life expectancy? They have to say ‘well, in this disease there is no way to guess life expectancy, we’ve seen people in almost a comatose stage, certainly a vegetative stage, live for as long as 20 years’. And that makes the heart of a spouse really sink because you think, ‘well, I’ll be old myself then and what is there left really in life …’. (Carer)^36^ ‘None of his doctors know anything about his condition. I read all of the research and I inform his doctors because [Person with dementia]’s doctors don’t have any other patients with this illness, and honestly they don’t seem very interested’. (Carer)^31^	4, 31–33, 36, 42, 47, 59, 62, 66, 68, 72, 74, 77, 79, 82, 83, 85, 87–89, 91, 94, 96
Making uninformed choices	‘I actually think that before [clinicians] have family members fill out that [do-not-hospitalise] form, somebody should actually sit down and explain every little thing on the form, instead of, “do you want to do this, do you want to do that.” They’re doing it in a rush, and you don’t know half the time what you’re signing’. (Carer)^30^ ‘[I want] to know when it is really appropriate to access services from hospice and when it may not be. Knowing how to ask the right questions and who to ask them of, created a lot of turmoil for me, like “my mother is going to die in six months, holy cow!”’. (Carer)^75^	4, 28, 30, 33, 38, 48, 54, 61, 63, 67, 68, 71, 74–76, 79, 87, 88, 94, 98
Deprived of hospice access and support at end of life	‘Nobody came to us soon enough to consider hospice, which, at the end, was really sad’. (Carer)^28^ ‘I basically spent a week fooling myself, thinking that I could find Mother decent care without bankrupting us’. (Carer)^44^ ‘She was on home hospice, but actually she got kicked out of home hospice, because she was being cared for pretty well. I say she was “kicked out” of hospice, they say she “graduated” from hospice’. (Carer)^28^‘How come you don’t think she’s for hospice? She’s just a body lying there – doesn’t talk, doesn’t change herself, doesn’t feed herself, doesn’t do anything for herself. She’s literally just lying in a hospital bed/ We need help, and, day-by-day – she could be gone tomorrow. We couldn’t imagine that [hospice] would let us go’. (Carer)^63^	4, 28, 39, 59, 62, 63, 89,96

Quotations are from study participants; the codebook containing the themes and sections from each study coded to the respective themes are available on requisition from corresponding author.

a Studies which contributed data to the given subtheme.

### Avoiding dehumanising treatment and care

#### Remaining connected

Nearing death, people with dementia and their carers highlighted a need for social, sensory and spiritual engagement consistent with a ‘normal life’^57^ without dementia. One person with dementia described spiritual engagement as attending to the ‘true self’^23^ who has a meaningful existence after memory, communication and emotional expression have diminished. As dementia was seen to ‘take away’^73^ the person they once knew, carers believed it was important that the person with dementia continue to participate in recreational activities, spend time with family and be treated as an individual with a unique personality. For instance, one carer felt it was important that her husband be surrounded by his own belongings while living in a nursing home. Some carers reflected on how having nursing staff physically present and verbally communicative with their dying relative, even when the person with dementia could no longer communicate, had inspired their trust and relieved anxiety.

#### Delaying institutionalisation

Some carers were upset because they perceived treatment and care delivered in nursing facilities or hospitals had ‘robbed’^24^ the person with dementia of dignity at end of life. Some carers felt ACP preferences had been deliberately ignored by care staff or were not prioritised within these services because all people with dementia were seen to be treated the same ‘based on institutionalised care practices’.^25^ In the absence of knowing what the person with dementia would have preferred and because of a perception that institutionalised care would result in undignified care, some carers decided to keep the person with dementia at home as long as possible or endeavoured to provide care themselves (such as dressing and showering), to avoid ‘humiliating’^81^ acts of care. People with dementia and carers regarded dignified care within institutions as having preferences known and adhered to by health professionals, maintaining a respectable physical appearance, having privacy, being clothed, having hygiene maintained, being comfortable, being able to use the toilet and being free of fear and pain.

#### Rejecting the burdens of futile treatment

Some carers who had participated in ACP perceived dementia as a terminal condition and focused on promoting a ‘good death’^58^ in line with the preferences of the person with dementia only. Other carers reflected on circumstances in which they believed aggressive treatment caused only prolonged suffering and, thus, were preferring ‘to let nature take its course’^26^ – to have a ‘natural death without machines’.^59^ In contrast, some carers who had not participated in ACP struggled to understand dementia’s dying trajectory and felt obliged to treat what could be treated (e.g. pneumonia, urinary tract infection, loss of swallowing), even in the advanced stages.

### Confronting emotionally difficult conversations

#### Signifying death

Some carers felt unable to discuss ACP and end-of-life issues with the person with dementia as they were concerned it would cause the person with dementia to ‘feel [they were] dying’.^5^ Some were reluctant to set goals or document ACP preferences (such as to refuse resuscitation) because they felt such a process was too harsh or inhumane and might be perceived as ‘putting [the person with dementia] to [their] death’.^4^

#### Unpreparedness to face impending cognitive decline

Some people with dementia and their carers made an active decision not to participate in ACP, as they found the progressive and inevitable cognitive deterioration associated with dementia too ‘frightening’^60^ to think about in advance. Some felt there was ‘no real urgency’^4^ for ACP and avoided the discussion until the timing felt right. One carer reflected that they had ‘procrastinated for years’^26^ to avoid ACP until the person with dementia was ‘right on the verge’ of losing the ability to communicate. Other carers regretted that they had not completed ACP earlier because they felt the person with dementia’s illness had advanced too far for them to be able to participate in the decision-making process.

#### Locked into a pathway

Some people with dementia and carers feared that completing ACP documentation may be overly binding and lock the person with dementia into a ‘static and immutable’^27^ contract. ACP documents were perceived by some to be a barrier to autonomy and they expressed concern that they were just ‘tick[ing] certain boxes’^24^ on a pathway predefined by health professionals. Thus, some preferred to make informal advance care plans, believing that substitute decision-makers would more accurately communicate the preferences of the person with dementia at end of life. In contrast, some carers and people with dementia who had participated in ACP felt reassured because they perceived ACP documentation could be revised later if the preferences of the person with dementia changed.

### Navigating existential tensions

#### Accepting inevitable incapacity and death

People with dementia and their carers who were more accepting of the progressive, ‘irreversible’^82^ and terminal nature of dementia appeared better able to engage in ACP discussions than those who felt unprepared to face these issues. ACP assisted some carers to confront fears of losing their loved one by preparing them for death and by enabling them to feel more comfortable making end-of-life treatment decisions regarding resuscitation and artificial nourishment. Some carers believed that a lack of communication with health professionals implicitly reinforced beliefs among people with dementia and/or their carers that death should remain unspoken. Thus, they felt having supportive health professionals who encouraged discussions of death was integral to reaching this acceptance.

#### Fear of being responsible for cause of death

Even when ACP had occurred, some carers were deeply conflicted when called upon to make end-of-life decisions. The main source of difficulty appeared to be operationalising goals for comfort into actions, such as denying hospitalisation or withholding nutrition or fluids, because such actions would effectively ‘cause’^61^ the death of their loved one. Thus, once carers realised that adhering to the operationalisation of the ACP preferences of the person with dementia would lead to the person’s death, they became less able to follow the preferences as they felt responsible for ‘killing’^28^ the person with dementia. This proved too overwhelming for some and led them to ignore the preferences of the person with dementia against life-sustaining treatments in order to preserve their own peace of mind and maintain a clear ‘conscience’.

#### Alleviating decisional responsibility

ACP helped some carers to overcome the perceived guilt and ‘burden’^29^ that they believed they would have otherwise experienced during end-of-life decision-making; this was particularly notable among carers who were adhering to ACP preferences of the person with dementia to forego life-sustaining treatments. Nonetheless, carers expressed a need to frame decision-making from the perspective of the person with dementia, such that documented advance care plans became ‘a bible’^30^ for decision-making and that their responsibility was simply to ‘implement [decisions]’^98^ or to ‘fulfil [the person with dementia’s] wishes’.^30^

### Defining personal autonomy

#### Struggling with unknown preferences

Carers were confronted by a ‘moral dilemma’^93^ or ‘quandary’^26^ when a medical decision was required and an advance care plan had not been made. Under these circumstances, carers relied on their knowledge and sense of who the person was before the onset of dementia to make treatment and care decisions. However, some carers found these decisions were accompanied by feelings of ambivalence and guilt and left them to hope that they were doing the ‘right thing’^14^ for the person with dementia. Such circumstances appeared easier for spousal carers who felt that being ‘married so many years’^26^ to the person with dementia led to an implicit understanding of their preferences; but more difficult for carers who had shared less frequent contact, such as children or extended relatives.

#### Depending on carer advocacy

Even when an advance care plan had been documented, some people with dementia expected their carers to be present during medical decisions, to protect and enforce their end-of-life preferences. Similarly, some carers felt it was their duty to ensure that the preferences of the person with dementia were adhered to. For example, such carers believed doctors were more focused on ‘meeting targets’^3^ and ensuring that nobody would ‘die on their watch’.^31^ Thus, they anticipated that they might ‘have to fight the doctors’^59^ to prevent unwanted interventions, such as inserting a feeding tube or central line. One carer felt they had failed to advocate for the person with dementia because they had been ‘pressured’^31^ by doctors to consent to the use of life-sustaining treatments, despite knowing this was in conflict with the person’s ACP preferences.

#### Justifying treatments for health deteriorations

Even when ACP had occurred, some carers felt they needed to ‘breach’ the preferences of the person with dementia to withhold life-prolonging treatments, in order to save them from a ‘premature’ death. Carers who perceived health events, such as colds, bone fractures, pneumonia and dysphagia, as ‘curable’ with ‘modern medicine’, appeared to discount the life-threatening nature of such episodes in dementia. Rather, they perceived treatments involving minor surgery, antibiotics and feeding tubes as low risk and likely to return the person with dementia back to their previous state of health.

### Lacking confidence in healthcare settings

#### Distrusting clinician’s mastery and knowledge of dementia

Some carers perceived a degree of ‘medical uncertainty’ among healthcare providers, leading them to feel frustrated and lose trust in healthcare providers. Such carers reflected on perceived inaccuracy at the point of diagnosis or when seeking advice about prognosis and/or treatment options for the person with dementia. In addition, some believed that physicians had purposefully disengaged from the person with dementia and their carers in conversations about ACP because they lacked confidence in making clinical judgements and the ACP process. Others felt that healthcare providers had actively ‘ignored’^74^ the carer’s concerns about the cognitive deterioration of the person with dementia and their inability to obtain a firm diagnosis was a barrier to them being able to plan for the future. Nonetheless, some carers looked to healthcare providers as the ‘specialists’^32^ of dementia and some carers chose not to ‘question [clinicians] decisions or actions about care’.^62^

#### Making uninformed choices

Some carers who had completed ACP on behalf of a non-competent person with dementia felt that they had not made the best advance care plan, as they had not been given enough time or received enough support from clinicians to explore all options. One carer reflected that they could have ‘come up with a better plan’^75^ had they been better informed of the course of dementia and treatment options at the time and one carer who had completed a ‘Do-Not-Hospitalise’ form reflected that it had been completed in a rush and that they ‘[didn’t] know … what [they were] signing’.^30^ Both people with dementia and carers expressed a need for ‘better education’ around the course of dementia and medical decisions they were likely to face to enable them to participate meaningfully in ACP, such as ‘what a feeding tube is [and] what a DNR [do not resuscitate] is …’,^33^ and for communication to be ongoing and revisited to allow time to digest the relevant information.

#### Deprived of access to hospice care and support at end of life

Overall, carers were disappointed by difficulties they experienced ‘getting through the front lines’^28^ and accessing hospice and support when the person with dementia was approaching end of life, which they believed was a result of limited care options, high costs of services and inconsistent/lack of communication from health providers. Some carers felt ‘cheated’^28^ because the person with dementia was not offered hospice until it was too late to consider or benefit from the care. For others who had accessed hospice, some later became overwhelmed when the person with dementia was discharged or ‘kicked out of hospice’^28^ despite being considered ‘terminal’ but ‘not dying fast enough’^63^ to qualify for hospice care. Thus, carers believed the provision and duration of hospice access was inadequate in dementia and some questioned the appropriateness of using an ‘end-of-life care’ model in dementia given its unpredictable disease trajectory.

## Discussion

People with dementia and their carers felt uncertain in making treatment decisions in the context of ACP and end-of-life care. They had to confront emotionally difficult conversations and navigate existential tensions during ACP; while also feeling a sense of distrust and a lack of confidence in the clinical information and support available to them in healthcare settings. Because of this, some were reluctant to discuss ACP preferences as they felt that ACP signified impending death; were unprepared to face the inevitable cognitive deterioration; or feared that by completing an advance care plan, they would be locked into a predefined pathway for care. In addition, carers needed to overcome uncertainty in decision-making if the person with dementia had not previously expressed their preferences; they felt adhering to the ACP preferences of the person with dementia would make them responsible for the person’s death; or they experienced disagreement with clinicians when advocating for the preferences of the person with dementia. Overall, people with dementia and their carers appeared more willing and prepared to undertake ACP if they were more accepting of the progressive, irreversible and terminal nature of dementia, and viewed ACP as a flexible and ongoing discussion with supportive healthcare providers.

Our review found that even when ACP documentation had been completed, some carers felt unprepared for making end-of-life decisions for the person with dementia. For some, this may have resulted from insufficient consideration of the types of decisions typically encountered through the dementia illness trajectory. It also acknowledges that uncertainty may be inevitable in some contexts of dementia and substitute decision-makers may require some ‘leeway’ when adhering to ACP preferences.^100^ In our study, some carers struggled with decisions to refuse or restrict interventions, because they felt a level of personal responsibility for the death of the person with dementia. In addition, some felt they had breached the ACP preference of the person with dementia to refuse life-sustaining treatments when health complications arose that they perceived as curable. Nonetheless, some carers, who viewed themselves as only a messenger for the preferences of the person with dementia, expressed relief because they felt they had been spared from having to make otherwise burdensome end-of-life decisions.

The challenges to achieving person-centred care for people with advanced dementia, particularly in institutionalised settings, have been well described in the literature spanning different healthcare settings.^101^ People with dementia and carers perceive a lack of personalised care, inclusion or choice in healthcare decisions and health professionals missing opportunities to enhance physical and psychological comfort.^102,103^ The themes identified in this review, such as avoiding dehumanising treatment and care and defining personal autonomy, similarly emphasise person-centred care as a central goal and priority of dementia care. For example, in this review, people with dementia and their carers expressed a need to maintain a connection to a ‘normal life’ and regarded dignified care as having preferences known and adhered to by health professionals. However, some carers experienced difficulties collaborating and communicating with health professionals, such as obtaining accurate information about prognosis or treatment options for the person with dementia or accessing hospice services or support at end of life.

While ACP has potential benefits for people with dementia and carers, implementing systems and structures to support ACP in dementia is complex. One challenge is that in dementia, cognition and decision-making capacity deteriorate and, in some situations, result in a lack of ability to understand the concepts involved in ACP.^24,104,105^ In addition, the preferences specified by a person during ACP may not cover all care decisions or daily care activities in advanced dementia, whereby a person’s ability to communicate needs is restricted, both by their own communication impairments (e.g. loss of speech) and by the health professional’s ability to assess and recognise the person’s needs and symptoms. Furthermore, the diagnosis of dementia may only occur years after the disease has begun,^94,106^ leaving decision-making responsibility up to a substitute decision-maker if the person is unable to participate themselves. Our new thematic schema of the perspectives of people with dementia and carers on ACP and end-of-life care draws attention to several key challenges: carers’ distrust in clinicians’ ability to provide accurate diagnosis and advice about the prognosis of dementia; difficulties facing and accepting cognitive decline and approaching death; uncertainties in defining and adhering to ACP preferences of the person with dementia; and barriers in accessing hospice and support at end of life. Our synthesis also highlights carers’ beliefs that using an ‘end-of-life care’ model in dementia is problematic given the dimensions of uncertainty experienced by carers across the illness trajectory.

Our review reflects findings from previous studies examining perspectives on ACP and end-of-life care among people with other chronic and progressive illnesses such as chronic kidney disease, chronic obstructive pulmonary disease and cardiac failure. Studies among these populations have also documented perceptions that ACP signifies death^107^ or that completing an advance care plan may be overly binding.^108^ Frustration about being unable to obtain a clear prognosis and access palliative care services has also been reported.^109^ In addition, ACP and end-of-life decision-making can require negotiation with existential tensions when deciding to commence or continue with life-sustaining treatment.^110^ Similarly, our review identified that some people with dementia and their carers found ACP emotionally difficult, distrusted clinicians’ ability to diagnose and prognosticate in dementia, and felt deprived of palliative care services. Moreover, carers needed to overcome existential concerns in dementia, particularly when the person with dementia lost capacity to make decisions for themselves.

### Strengths and limitations

In this review, we conducted a comprehensive search and independent assessment of study reporting; and synthesised data from different healthcare contexts where people with dementia are likely to undertake ACP (such as care home, community, hospital and hospice) to develop a new and comprehensive thematic framework. However, there are some potential limitations. Only one in five of the included studies reported whether data saturation was reached, which questions whether subsequent data collection would have identified additional or novel concepts in those studies. In addition, less than one-third of the studies included people with dementia, and this may reflect the challenges of involving people with dementia in qualitative studies,^111^ and the need to address these. Additional concepts regarding the perspectives of people with dementia on ACP and end-of-life care may have been identified if there had been a greater number of qualitative studies including people with dementia. We excluded articles that were not published in English and the majority of studies were from high-income English-speaking countries; thus, the transferability of the findings beyond these settings and populations is unclear. Nonetheless, the analytical themes offer a high-level conceptual framework regarding ACP and end-of-life care that may be applicable across different contexts.

### Implications for policy, future practice and research

In ACP and end-of-life care in dementia, we suggest that health professionals demonstrate empathy and attend to people’s uncertainty in decision-making. Models of ACP that appear to lock individuals into a pathway, or do not facilitate acceptance of the natural course of dementia, that is, the cognitive decline and eventual mortality, may fail to elicit healthcare preferences before the person with dementia loses capacity. Thus, strategies to improve clinicians’ mastery and knowledge of palliative and dementia care, with respect to discussions about prognosis and treatment and care options available now and in the future, are essential to increasing confidence among people with dementia and their carers as they navigate the healthcare system. Moreover, consistent with past recommendations,^100^ future ACP strategies should focus on preparing substitute decision-makers for potential uncertainties that may arise ‘in-the-moment’ when adhering to the person with dementia’s ACP preferences and plan for some leeway in these circumstances.

We suggest future research aims to further describe the perspectives of people with dementia on ACP and to consider addressing the challenges of conducting qualitative interviews with people experiencing varying levels of cognitive decline. Strategies to optimise participation of people with dementia in such research may include scheduling interviews with people when they are usually most alert during the day; being flexible in communication style and restructuring questions if they are not understood initially; and supplementing interviews with other qualitative techniques, such as observation.^112,113^ Furthermore, in general medical settings, ACP has previously been shown to improve the likelihood that preferences will be known and adhered to at end of life and reduce stress, anxiety and depression among surviving relatives.^114^ However, similar high-quality studies of ACP have not yet been conducted in people with dementia^115^ and thus future randomised controlled trials of ACP are needed to further understand the impact of ACP on people with dementia and their carers.

## Conclusion

For people with dementia and their carers, the experience of ACP and end-of-life care was characterised by a sense of uncertainty in decision-making. ACP required some to confront emotionally difficult conversations and some carers felt unprepared in being able to adhere to ACP preferences and make end-of-life decisions on behalf of the person with dementia. We suggest health professionals demonstrate empathy and aim to facilitate acceptance of the inevitable cognitive decline and death in dementia and provide an understanding of the decisions that may need to be made along the trajectory of dementia. In addition, future ACP strategies should attend to potential uncertainties that may arise when carers are attempting to adhere to the person with dementia’s ACP preferences at end of life.

## Supplemental Material

809571_supp_mat – Supplemental material for Perspectives of people with dementia and carers on advance care planning and end-of-life care: A systematic review and thematic synthesis of qualitative studies Click here for additional data file. Supplemental material, 809571_supp_mat for Perspectives of people with dementia and carers on advance care planning and end-of-life care: A systematic review and thematic synthesis of qualitative studies by Marcus Sellars, Olivia Chung, Linda Nolte, Allison Tong, Dimity Pond, Deirdre Fetherstonhaugh, Fran McInerney, Craig Sinclair and Karen M Detering in Palliative Medicine

## References

[1] World Health Organization. The epidemiology and impact of dementia: current state and future trends. Geneva: World Health Organization, 2015, http://www.who.int/mental_health/neurology/dementia/dementia_thematicbrief_epidemiology.pdf (accessed 16 January 2018).

[2] Downs M Bowers B. Excellence in dementia care: research into practice. Maidenhead: McGraw-Hill Education, 2014.

[3] Dening KH King M Jones L et al Healthcare decision-making: past present and future, in light of a diagnosis of dementia. Int J Palliat Nurs 2017; 23(1): 4–11. 28132606 10.12968/ijpn.2017.23.1.4

[4] Dickinson C Bamford C Exley C et al Planning for tomorrow whilst living for today: the views of people with dementia and their families on advance care planning. Int Psychogeriatr 2013; 25(12): 2011–2021. 24053783 10.1017/S1041610213001531

[5] Fetherstonhaugh D McAuliffe L Bauer M et al Decision-making on behalf of people living with dementia: how do surrogate decision-makers decide? J Med Ethics 2017; 43(1): 35–40. 27780889 10.1136/medethics-2015-103301

[6] Australian Commission on Safety Quality in Health Care. National consensus statement: essential elements for safe and high-quality end-of-life care. Sydney, NSW, Australia: ACSQHC, https://www.safetyandquality.gov.au/wp-content/uploads/2015/05/National-Consensus-Statement-Essential-Elements-forsafe-high-quality-end-of-life-care.pdf (2015, accessed 16 January 2018).

[7] Sudore RL Lum HD You JJ et al Defining advance care planning for adults: a consensus definition from a multidisciplinary Delphi panel. J Pain Symptom Manage 2017; 53(5): 821.e1–832.e1. 28062339 10.1016/j.jpainsymman.2016.12.331 PMC5728651

[8] Mitchell SL Kiely DK Hamel MB. Dying with advanced dementia in the nursing home. Arch Intern Med 2004; 164(3): 321–326. 14769629 10.1001/archinte.164.3.321

[9] Vandervoort A van den Block L van der Steen JT et al Advance directives and physicians’ orders in nursing home residents with dementia in Flanders, Belgium: prevalence and associated outcomes. Int Psychogeriatr 2012; 24(7): 1133–1143. 22364648 10.1017/S1041610212000142

[10] Garand L Dew MA Lingler JH et al Incidence and predictors of advance care planning among persons with cognitive impairment. Am J Geriatr Psychiatry 2011; 19(8): 712–720. 21785291 10.1097/JGP.0b013e3181faebef PMC3145957

[11] Davies N Maio L Rait G et al Quality end-of-life care for dementia: what have family carers told us so far? A narrative synthesis. Palliat Med 2014; 28(7): 919–930. 24625567 10.1177/0269216314526766 PMC4232347

[12] Evers MM Purohit D Perl D et al Palliative and aggressive end-of-life care for patients with dementia. Psychiatr Serv 2002; 53: 609–613. 11986512 10.1176/appi.ps.53.5.609

[13] McCarthy M Addington-Hall J Altmann D. The experience of dying with dementia: a retrospective study. Int J Geriatr Psychiatry 1997; 12(3): 404–409. 9152728

[14] Ashton SE Roe B Jack B et al End of life care: the experiences of advance care planning amongst family caregivers of people with advanced dementia: a qualitative study. Dementia 2016; 15(5): 958–975. 25187482 10.1177/1471301214548521

[15] Poppe M Burleigh S Banerjee S. Qualitative evaluation of advanced care planning in early dementia (ACP-ED). PLoS ONE 2013; 8(4): e60412. 23630571 10.1371/journal.pone.0060412 PMC3629937

[16] Van Soest-Poortvliet MC van der Steen JT Gutschow G et al Advance care planning in nursing home patients with dementia: a qualitative interview study among family and professional caregivers. J Am Med Direct Assoc 2015; 16(11): 979–989. 10.1016/j.jamda.2015.06.015 26255099

[17] De Boer ME Dröes R-M Jonker C et al Thoughts on the future: the perspectives of elderly people with early-stage Alzheimer’s disease and the implications for advance care planning. AJOB Prim Res 2012; 3(1): 14–22.

[18] Liamputtong P. Qualitative research methods. South Melbourne, Vic : Oxford University Press, 2012.

[19] Braun V Clarke V. Using thematic analysis in psychology. Qual Res Psychol 2006; 3(2): 77–101.

[20] Thomas J Harden A. Methods for the thematic synthesis of qualitative research in systematic reviews. BMC Med Res Methodol 2008; 8(1): 45. 18616818 10.1186/1471-2288-8-45 PMC2478656

[21] Tong A Flemming K McInnes E et al Enhancing transparency in reporting the synthesis of qualitative research: ENTREQ. BMC Med Res Methodol 2012; 12(1): 181. 23185978 10.1186/1471-2288-12-181 PMC3552766

[22] Tong A Sainsbury P Craig J. Consolidated criteria for reporting qualitative research (COREQ): a 32-item checklist for interviews and focus groups. Int J Qual Health Care 2007; 19(6): 349–357. 17872937 10.1093/intqhc/mzm042

[23] Fleming R Kelly F Stillfried G. ‘I want to feel at home’: establishing what aspects of environmental design are important to people with dementia nearing the end of life palliative care in other conditions. BMC Palliat Care 2015; 14(1): 26. 25962895 10.1186/s12904-015-0026-y PMC4436026

[24] Dening KH Jones L Sampson EL. Preferences for end-of-life care: a nominal group study of people with dementia and their family carers. Palliat Med 2013; 27(5): 409–417. 23128905 10.1177/0269216312464094 PMC3652642

[25] Rosemond C Hanson LC Zimmerman S. Goals of care or goals of trust? How family members perceive goals for dying nursing home residents. J Palliat Med 2017; 20(4): 360–365. 27898281 10.1089/jpm.2016.0271 PMC5385445

[26] Black BS Fogarty LA Phillips H et al Surrogate decision makers’ understanding of dementia patients’ prior wishes for end-of-life care. J Aging Health 2009; 21(4): 627–650. 19269928 10.1177/0898264309333316 PMC2832743

[27] Saini G Sampson EL Davis S et al An ethnographic study of strategies to support discussions with family members on end-of-life care for people with advanced dementia in nursing homes. BMC Palliat Care 2016; 15: 55. 27388766 10.1186/s12904-016-0127-2 PMC4936120

[28] Lewis LF. Caregivers’ experiences seeking hospice care for loved ones with dementia. Qual Health Res 2014; 24(9): 1221–1231. 25079503 10.1177/1049732314545888

[29] Volicer L Stets K. Acceptability of an advance directive that limits food and liquids in advanced dementia. Am J Hosp Palliat Care 2016; 33(1): 55–63. 25313239 10.1177/1049909114554078

[30] Mann E Goff SL Colon-Cartagena W et al Do-not-hospitalize orders for individuals with advanced dementia: healthcare proxies’ perspectives. J Am Geriatr Soc 2013; 61(9): 1568–1573. 23888937 10.1111/jgs.12406 PMC3773256

[31] Noh H Kwak J. End-of-life decision making for persons with dementia: proxies’ perception of support. Dementia 2018; 17: 478–493. 27165983 10.1177/1471301216648473

[32] Wakunami M Kawabata H Murakami M et al Families’ acceptance of near death: a qualitative study of the process for introducing end-of-life care. Geriatr Gerontol Int 2009; 9(2): 140–147. 19740357 10.1111/j.1447-0594.2008.00494.x

[33] Givens JL Lopez RP Mazor KM et al Sources of stress for family members of nursing home residents with advanced dementia. Alz Dis Assoc Dis 2012; 26(3): 254–259. 10.1097/WAD.0b013e31823899e4 PMC3288670 22037596

[34] Beernaert K Deliens L De Vleminck A et al Is there a need for early palliative care in patients with life-limiting illnesses? Interview study with patients about experienced care needs from diagnosis onward. Am J Hosp Palliat Med 2016; 33(5): 489–497. 10.1177/1049909115577352 25852203

[35] Beernaert K Van den Block L Van Thienen K et al Family physicians’ role in palliative care throughout the care continuum: stakeholder perspectives. Fam Pract 2015; 32(6): 694–700. 26373666 10.1093/fampra/cmv072

[36] Bonnel WB. Not gone and not forgotten: a spouse’s experience of late-stage Alzheimer’s disease. J Psychosoc Nurs Men 1996; 34(8): 23–40. 10.3928/0279-3695-19960801-14 8856601

[37] Cairns M. In sickness and in health: an exploration of some of the unconscious processes involved in the decision by family caregivers to place a family member with dementia in residential care. Psychoanal Psychother 2012; 26(1): 34–47.

[38] Forbes S Bern-Klug M Gessert C. End-of-life decision making for nursing home residents with dementia. J Nurs Scholarsh 2000; 32(3): 251–258. 12462819 10.1111/j.1547-5069.2000.00251.x

[39] Glass AP. Family caregiving and the site of care: four narratives about end-of-life care for individuals with dementia. J Soc Work End Life Palliat Care 2016; 12(1–2): 23–46. 27143572 10.1080/15524256.2016.1156605

[40] Goodman C Amador S Elmore N et al Preferences and priorities for ongoing and end-of-life care: a qualitative study of older people with dementia resident in care homes. Int J Nurs Stud 2013; 50(12): 1639–1647. 23866093 10.1016/j.ijnurstu.2013.06.008

[41] Groen-van de Ven L Smits C Span M et al The challenges of shared decision making in dementia care networks. Int Psychogeriatr 2016; 30(6): 1–15. 10.1017/S1041610216001381 27609338

[42] Hirschman KB Kapo JM Karlawish JH. Why doesn’t a family member of a person with advanced dementia use a substituted judgment when making a decision for that person? Am J Geriatr Psychiatry 2006; 14(8): 659–667. 16861370 10.1097/01.JGP.0000203179.94036.69

[43] Jox RJ Denke E Hamann J et al Surrogate decision making for patients with end-stage dementia. Int J Geriatr Psychiatry 2012; 27(10): 1045–1052. 22139621 10.1002/gps.2820

[44] Lewis LF. Caregiving for a loved one with dementia at the end of life. Am J Alzheimers Dis 2015; 30(5): 488–496. 10.1177/1533317514559829 PMC10852728 25425737

[45] Pasman HRW The BAM Onwuteaka-Philipsen BD et al Participants in the decision making on artificial nutrition and hydration to demented nursing home patients: a qualitative study. J Aging Stud 2004; 18(3): 321–335.

[46] Peacock SC Hammond-Collins K Forbes DA. The journey with dementia from the perspective of bereaved family caregivers: a qualitative descriptive study. BMC Nurs 2014; 13(1): 42. 25435810 10.1186/s12912-014-0042-x PMC4247750

[47] Powers BA Watson NM. Meaning and practice of palliative care for nursing home residents with dementia at end of life. Am J Alzheimers Dis 2008; 23(4): 319–325. 10.1177/1533317508316682 PMC10697386 18453644

[48] Robinson EM. Wives’ struggle in living through treatment decisions for husbands with advanced Alzheimer’s disease. J Nurs Law 2000; 7(1): 21–39. 12545984

[49] Sanders S Butcher HK Swails P et al Portraits of caregivers of end-stage dementia patients receiving hospice care. Death Stud 2009; 33(6): 521–556. 19565686 10.1080/07481180902961161

[50] Sarabia-Cobo CM Pérez V de Lorena P et al Decisions at the end of life made by relatives of institutionalized patients with dementia. Appl Nurs Res 2016; 31: e6–e10. 26954489 10.1016/j.apnr.2016.02.003

[51] Slape J. Dementia and palliative care: the spiritual needs of family members. J Relig Spiritual Aging 2014; 26(2–3): 215–230.

[52] The AM Pasman R Onwuteaka-Philipsen B et al Withholding the artificial administration of fluids and food from elderly patients with dementia: ethnographic study. Br Med J 2002; 325(7376): 1326–1329. 12468479 10.1136/bmj.325.7376.1326 PMC137804

[53] Van der Steen JT Lemos Dekker N Gijsberts MJHE et al Palliative care for people with dementia in the terminal phase: a mixed-methods qualitative study to inform service development. BMC Palliat Care 2017; 16(1): 28. 28454534 10.1186/s12904-017-0201-4 PMC5410050

[54] Poole M Bamford C McLellan E et al End-of-life care: a qualitative study comparing the views of people with dementia and family carers. Palliat Med 2018; 32(3): 631–642. 29020864 10.1177/0269216317736033

[55] Andrews S McInerney F Toye C et al Knowledge of dementia: do family members understand dementia as a terminal condition? Dementia 2017; 16(5): 556–575. 26394629 10.1177/1471301215605630

[56] Lamahewa K Mathew R Iliffe S et al A qualitative study exploring the difficulties influencing decision making at the end of life for people with dementia. Health Expect 2018; 21(1): 118–127. 28640487 10.1111/hex.12593 PMC5750695

[57] Snyder EA Caprio AJ Wessell K et al Impact of a decision aid on surrogate decision-makers’ perceptions of feeding options for patients with dementia. J Am Med Direct Assoc 2013; 14(2): 114–118. 10.1016/j.jamda.2012.10.011 PMC3563876 23273855

[58] Stewart-Archer LA Afghani A Toye CM et al Dialogue on ideal end-of-life care for those with dementia. Am J Hosp Palliat Care 2015; 32(6): 620–630. 24782574 10.1177/1049909114532342

[59] Gessert CE Elliott BA Peden McAlpine C. Family decision-making for nursing home residents with dementia: rural-urban differences. J Rural Health 2006; 22(1): 1–8. 16441330 10.1111/j.1748-0361.2006.00013.x

[60] Boyd R. End-of-life care. Discussing end-of-life care with people with dementia: a word of caution. Ment Health Nurs 2011; 31(1): 14–17.

[61] Gessert CE Forbes S Bern-Klug M. Planning end-of-life care for patients with dementia: roles of families and health professionals. Omega 2000; 42(4): 273–291. 12569923 10.2190/2mt2-5gyu-gxvv-95ne

[62] Shuter P Beattie E Edwards H. An exploratory study of grief and health-related quality of life for caregivers of people with dementia. Am J Alzheimers Dis 2014; 29(4): 379–385. 10.1177/1533317513517034 PMC10852965 24381138

[63] Wladkowski SP. Live discharge from hospice and the grief experience of dementia caregivers. J Soc Work End Life Palliat Care 2016; 12(1/2): 47–62. 27143573 10.1080/15524256.2016.1156600

[64] Bosek MSD Lowry E Lindeman DA et al Promoting a good death for persons with dementia in nursing facilities: family caregivers’ perspectives. Jonas Healthc Law Ethic Regul 2003; 5(2): 34–41. 10.1097/00128488-200306000-00006 12789031

[65] Cronfalk BS Ternestedt B-M Norberg A. Being a close family member of a person with dementia living in a nursing home. J Clin Nurs 2017; 26: 3519–3528. 28042920 10.1111/jocn.13718

[66] De Vries K Sque M Bryan K et al Variant Creutzfeldt-Jakob disease: need for mental health and palliative care team collaboration. Int J Palliat Nurs 2003; 9(12): 512–520. 14765007 10.12968/ijpn.2003.9.12.11992

[67] Juozapavicius KP Weber JA. A reflective study of Alzheimer’s caregivers. Am J Alzheimers Dis 2001; 16(1): 11–20. 10.1177/153331750101600108 PMC10832635 11416944

[68] Muders P Zahrt-Omar CA Bussmann S et al Support for families of patients dying with dementia: a qualitative analysis of bereaved family members’ experiences and suggestions. Palliat Support Care 2015; 13(3): 435–442. 24524412 10.1017/S1478951513001107

[69] Peacock S Duggleby W Koop P. The lived experience of family caregivers who provided end-of-life care to persons with advanced dementia. Palliat Support Care 2014; 12(2): 117–126. 23510738 10.1017/S1478951512001034

[70] Shanley C Fetherstonhaugh D McAuliffe L et al Providing support to surrogate decision-makers for people living with dementia: healthcare professional, organisational and community responsibilities. Health Soc Care Community 2017; 25: 1563–1570. 28569431 10.1111/hsc.12456

[71] Stirling C McLnerney F Andrews S et al A tool to aid talking about dementia and dying–development and evaluation. Collegian 2014; 21(4): 337–343. 25632731 10.1016/j.colegn.2013.08.002

[72] Tarter R Demiris G Pike K et al Pain in hospice patients with dementia: the informal caregiver experience. Am J Alzheimers Dis 2016; 31(6): 524–529. 10.1177/1533317516653825 PMC4982799 27303062

[73] Davies N Rait G Maio L et al Family caregivers’ conceptualisation of quality end-of-life care for people with dementia: a qualitative study. Palliat Med 2017; 31(8): 726–733. 27815555 10.1177/0269216316673552 PMC5625846

[74] Beisecker AE Chrisman SK Wright LJ. Perceptions of family caregivers of persons with Alzheimer’s disease: communication with physicians. Am J Alzheimers Dis 1997; 12(2): 73–83.

[75] Samia LW Hepburn K Nichols L. ‘Flying by the seat of our pants’: what dementia family caregivers want in an advanced caregiver training program. Res Nurs Health 2012; 35(6): 598–609. 22911130 10.1002/nur.21504

[76] Almberg BE Grafström M Winblad B. Caregivers of relatives with dementia: experiences encompassing social support and bereavement. Aging Ment Health 2000; 4(1): 82–89.

[77] Cahill S Doran D Watson M. Guidelines for nursing homes delivering end-of-life care to residents with dementia across the island of Ireland. Qual Ageing Old Adult 2012; 13(1): 60–70.

[78] Groen-van de Ven L Smits C Oldewarris K et al Decision trajectories in dementia care networks: decisions and related key events. Res Aging 2017; 39: 1039–1071. 27401681 10.1177/0164027516656741

[79] Livingston G Leavey G Manela M et al Making decisions for people with dementia who lack capacity: qualitative study of family carers in UK. BMJ 2010; 341: c4184. 20719843 10.1136/bmj.c4184 PMC2923693

[80] Mulqueen K Coffey A. Preferences of residents with dementia for end of life care. Nurs Old People 2017; 29(2): 26–30. 10.7748/nop.2017.e862 28244346

[81] Russell C Middleton H Shanley C. Dying with dementia: the views of family caregivers about quality of life. Aust J Ageing 2008; 27(2): 89–92. 10.1111/j.1741-6612.2008.00282.x 18713199

[82] Caron CD Griffith J Arcand M. Decision making at the end of life in dementia: how family caregivers perceive their interactions with health care providers in long-term-care settings. J Appl Gerontol 2005; 24(3): 231–247.

[83] Caron CD Griffith J Arcand M. End-of-life decision making in dementia: the perspective of family caregivers. Dementia 2005; 4(1): 113–136.

[84] Crowther J Wilson KCM Horton S et al Compassion in healthcare – lessons from a qualitative study of the end of life care of people with dementia. J Roy Soc Med 2013; 106(12): 492–497. 24108538 10.1177/0141076813503593 PMC3842856

[85] Forbes DA Finkelstein S Blake CM et al Knowledge exchange throughout the dementia care journey by Canadian rural community-based health care practitioners, persons with dementia, and their care partners: an interpretive descriptive study. Rural Remote Health 2012; 12(4): 2201. 23176308

[86] Godwin B Waters H. ‘In solitary confinement’: planning end-of-life well-being with people with advanced dementia, their family and professional carers. Mortality 2009; 14(3): 265–285.

[87] Jennings LA Palimaru A Corona MG et al Patient and caregiver goals for dementia care. Qual Life Res 2017; 26(3): 685–693. 28000094 10.1007/s11136-016-1471-7 PMC5382930

[88] Thuné-Boyle IC Sampson EL Jones L et al Challenges to improving end of life care of people with advanced dementia in the UK. Dementia 2010; 9(2): 259–284.

[89] Treloar A Crugel M Adamis D. Palliative and end of life care of dementia at home is feasible and rewarding: results from the ‘Hope for Home’ study. Dementia 2009; 8(3): 335–347.

[90] Stewart F Goddard C Schiff R et al Advanced care planning in care homes for older people: a qualitative study of the views of care staff and families. Age Ageing 2011; 40(3): 330–335. 21345840 10.1093/ageing/afr006

[91] Gil E Agmon M Hirsch A et al Dilemmas for guardians of advanced dementia patients regarding tube feeding. Age Ageing 2018; 47(1): 138–143. 29040344 10.1093/ageing/afx161

[92] Albinsson L Strang P. Existential concerns of families of late-stage dementia patients: questions of freedom, choices, isolation, death, and meaning. J Palliat Med 2003; 6(2): 225–235. 12854939 10.1089/109662103764978470

[93] Lawrence V Samsi K Murray J et al Dying well with dementia: qualitative examination of end-of-life care. Br J Psychiatr 2011; 199(5): 417–422. 10.1192/bjp.bp.111.093989 21947653

[94] Dening KH Greenish W Jones L et al Barriers to providing end-of-life care for people with dementia: a whole-system qualitative study. BMJ Support Palliat Care 2012; 2(2): 103–107. 10.1136/bmjspcare-2011-000178 24654049

[95] Hirschman KB Kapo JM Karlawish JH et al Identifying the factors that facilitate or hinder advance planning by persons with dementia. Alz Dis Assoc Dis 2008; 22(3): 293–298. 10.1097/WAD.0b013e318169d669 PMC2666935 18580595

[96] Hovland-Scafe CA Kramer BJ. Preparedness for death: how caregivers of elders with dementia define and perceive its value. Gerontologist 2017; 57: 1093–1102. 27342441 10.1093/geront/gnw092 PMC5881688

[97] Shanley C Russell C Middleton H et al Living through end-stage dementia: the experiences and expressed needs of family carers. Dementia 2011; 10(3): 325–340.

[98] Livingston G Lewis-Holmes E Pitfield C et al Improving the end-of-life for people with dementia living in a care home: an intervention study. Int Psychogeriatr 2013; 25(11): 1849–1858. 23924580 10.1017/S1041610213001221

[99] Hill SR Mason H Poole M et al What is important at the end of life for people with dementia? The views of people with dementia and their carers. Int J Geriatr Psychiatry 2017; 32: 1037–1045. 27515899 10.1002/gps.4564

[100] Sudore RL Fried TR. Redefining the ‘planning’ in advance care planning: preparing for end-of-life decision making. Ann Intern Med 2010; 153(4): 256. 20713793 10.1059/0003-4819-153-4-201008170-00008 PMC2935810

[101] Edvardsson D Winblad B Sandman P-O. Person-centred care of people with severe Alzheimer’s disease: current status and ways forward. Lancet Neurol 2008; 7(4): 362–367. 18339351 10.1016/S1474-4422(08)70063-2

[102] Clissett P Porock D Harwood RH et al The challenges of achieving person-centred care in acute hospitals: a qualitative study of people with dementia and their families. Int J Nurs Stud 2013; 50(11): 1495–1503. 23548170 10.1016/j.ijnurstu.2013.03.001

[103] McAllister CL Silverman MA. Community formation and community roles among persons with Alzheimer’s disease: a comparative study of experiences in a residential Alzheimer’s facility and a traditional nursing home. Qual Health Res 1999; 9(1): 65–85. 10558359 10.1177/104973299129121703

[104] Fazel S Hope T Jacoby R. Assessment of competence to complete advance directives: validation of a patient centred approach. BMJ 1999; 318(7182): 493–497. 10024254 10.1136/bmj.318.7182.493 PMC27742

[105] Gregory R Roked F Jones L et al Is the degree of cognitive impairment in patients with Alzheimer’s disease related to their capacity to appoint an enduring power of attorney? Age Ageing 2007; 36(5): 527–531. 17913758 10.1093/ageing/afm104

[106] Bradford A Kunik ME Schulz P et al Missed and delayed diagnosis of dementia in primary care: prevalence and contributing factors. Alz Dis Assoc Dis 2009; 23(4): 306. 10.1097/WAD.0b013e3181a6bebc PMC2787842 19568149

[107] Sellars M Clayton JM Morton RL et al An interview study of patient and caregiver perspectives on advance care planning in ESRD. Am J Kidney Dis 2018; 71: 216–224. 29132946 10.1053/j.ajkd.2017.07.021

[108] MacPherson A Walshe C O’Donnell V et al The views of patients with severe chronic obstructive pulmonary disease on advance care planning: a qualitative study. Palliat Med 2013; 27(3): 265–272. 22450158 10.1177/0269216312440606

[109] Murray SA Boyd K Kendall M et al Dying of lung cancer or cardiac failure: prospective qualitative interview study of patients and their carers in the community. BMJ 2002; 325(7370): 929. 12399341 10.1136/bmj.325.7370.929 PMC130056

[110] Tong A Cheung KL Nair SS et al Thematic synthesis of qualitative studies on patient and caregiver perspectives on end-of-life care in CKD. Am J Kidney Dis 2014; 63(6): 913–927. 24411716 10.1053/j.ajkd.2013.11.017

[111] Murphy K Jordan F Hunter A et al Articulating the strategies for maximising the inclusion of people with dementia in qualitative research studies. Dementia 2015; 14(6): 800–824. 24403314 10.1177/1471301213512489

[112] Beuscher L Grando VT. Challenges in conducting qualitative research with individuals with dementia. Res Gerontol Nurs 2009; 2(1): 6–11. 20077988 10.3928/19404921-20090101-04 PMC3076620

[113] Lloyd V Gatherer A Kalsy S. Conducting qualitative interview research with people with expressive language difficulties. Qual Health Res 2006; 16(10): 1386–1404. 17079800 10.1177/1049732306293846

[114] Detering KM Hancock AD Reade MC et al The impact of advance care planning on end of life care in elderly patients: randomised controlled trial. BMJ 2010; 340: c1345. 20332506 10.1136/bmj.c1345 PMC2844949

[115] Dixon J Karagiannidou M Knapp M. The effectiveness of advance care planning in improving end of life outcomes for people with dementia and their carers: a systematic review and critical discussion. J Pain Symptom Manage 2018; 55: 132–150. 28827062 10.1016/j.jpainsymman.2017.04.009

