# Supplementary material for: Perspectives of people with dementia and carers on advance care planning and end-of-life care: A systematic review and thematic synthesis of qualitative studies
Source: Palliat Med. 2018 Nov 8;33(3):274–90. doi: 10.1177/0269216318809571 (PMC6376607; doi:10.1177/0269216318809571)
Supplement: 809571_supp_mat – Supplemental material for Perspectives of people with dementia and carers on advance care planning and end-of-life care: A systematic review and thematic synthesis of qualitative studies [file 809571_supp_mat.pdf]

Supplementary Table S1. Search strategies

#	**MEDLINE 1946 to 6th July 2018**<br>Total search results = 629	**Embase 1947 to 6th July 2018**<br>Total search results = 948	**PsycINFO 1806 to 6th July 2018**<br>Total search results = 363	**CINAHL 1905 to 6th July 2018**<br>Total search results = 688
1	exp Adult/	exp Adult/	exp Patients/	exp Adult/
2	exp Patients/	exp Patients/	exp Caregivers/	exp Patients/
3	exp Caregivers/	exp Caregivers/	exp Family/	exp Caregivers/
4	exp Family/	exp Family/	exp Spouses/	exp Family/
5	exp Spouses/	exp Spouse/	exp Aging/	exp Spouses/
6	exp Aged/	exp Aged/	exp Guardianship/	exp Aged/
7	exp Legal Guardians/	exp Legal Guardians/	Client*.tw.	exp Guardianship, Legal/
8	Client*.tw.	exp Proxy/	Or/1-7	Client*.tw.
9	Or/1-8	Client*.tw.	exp Dementia/	Or/1-8
10	exp Dementia/	Or/1-9	exp Alzheimer's Disease/	exp Delirium, Dementia, Amnestic, Cognitive Disorders/
11	exp Neurocognitive Disorders/	exp Dementia/	Dement*.tw.	exp Dementia
12	Dement*.tw.	exp disorders of higher cerebral function	Alzheimer*.tw.	Dement*.tw.
13	Alzheimer*.tw.	Dement*.tw.	mild cognitive impair*.tw.	Alzheimer*.tw.
14	mild cognitive impair*.tw.	Alzheimer*.tw.	lewy bod*.tw.	mild cognitive impair*.tw.
15	lewy bod*.tw.	lewy bod*.tw.	vascular cognitive impair*.tw.	lewy bod*.tw.
16	vascular cognitive impair*.tw.	vascular cognitive impair*.tw.	Or/9-15	vascular cognitive impair*.tw.

17	Or/10-16	Or/11-16	8 and 16	Or/10-16
18	9 and 17	10 and 17	exp advance directives/	9 and 17
19	exp advance care planning/	exp living will/	Advance care plan*.tw.	exp decision making/
20	exp advance directives/	advance care plan*.tw.	living will*.tw.	Advance care plan*.tw.
21	living will*.tw.	advance directive*.tw.	exp decision making/	exp advance directives/
22	exp decision making/	exp decision making/	exp life-sustaining treatment/	life-sustaining treatment preference*.tw.
23	life-sustaining treatment preference*.tw.	exp life-sustaining treatment/	life-sustaining treatment preference*.tw.	exp Terminal Care
24	exp Terminal Care/	exp Terminal Care/	Terminal*.tw.	exp emergency treatment/
25	end-of-life*.tw.	end-of-life*.tw.	end-of-life*.tw.	end-of-life*
26	exp Attitude to Death/	exp Attitude to Death/	exp Death and Dying/	exp Attitude to Death/
27	exp Palliative care/	exp Palliative therapy/	exp Death Attitudes	exp Palliative care/
28	Or/19-27	Or/19-27	exp Palliative care/	Or/19-27
29	18 and 28	18 and 28	Or/18-28	18 and 28
30	exp qualitative research/	exp qualitative research/	17 and 29	exp qualitative studies/
31	Qualitative*.tw.	Qualitative*.tw.	exp qualitative methods/	Qualitative*.tw.
32	interview*.tw.	interview*.tw.	exp qualitative research/	interview*.tw.
33	focus group*.tw.	focus group*.tw.	Qualitative*.tw.	focus group*.tw.
34	thematic*.tw.	thematic*.tw.	interview*.tw.	thematic*.tw.
35	grounded theory.tw.	grounded theory.tw.	focus group*.tw.	grounded theory.tw.

36	phenomenol*.tw.	phenomenol*.tw.	thematic*.tw.	phenomenol*.tw.
37	content analysis.tw.	content analysis.tw.	grounded theory.tw.	content analysis.tw.
38	ethnograph*.tw.	ethnograph*.tw.	phenomenol*.tw.	ethnograph*.tw.
39	or/30-38	or/30-38	content analysis.tw.	or/30-38
40	29 and 39	29 and 39	ethnograph*.tw.	29 and 39
41			or/31-40	
42			30 and 41	

Supplementary Table S2. Characteristics of the Included Studies

Study	Country	No. of Person with dementia	Person with dementia age (mean [range])	No. of CG	CG age (mean [range])	Care Setting	Dementia Stage	Dementia Type	Methodology	Qualitative Data Collection	Analysis	Topic
Albinsson[1] (2011)	SE	-	-	20	[42-81]	Nursing home; daycare centre; private group dwelling	Moderately severe or severe (advanced-stage) dementia	Alzheimer's (n=12)	Qualitative design	Explorative interviews (face-to-face)	Hermeneutic approach	Existential concerns at End-of-Life
Almberg[2] (2000)	SE	-	-	30	71 [49-88]	Nursing home (institutionalised)	NS	Alzheimer's (n=53); Frontal temporal lobe (n=5); Lewy body (n=7); Parkinson's disease (n=1); Vascular (n=6); Wernicke-Korsakoff (n=1); Mixed (n=3); Unknown (n=8)	Qualitative design	Face-to-face interviews with open-ended questions	Content analysis	Bereavement and social support at End-of-Life
Andrews[3] (2017)	AU	-	-	10	NS	Dementia specific care unity	Advanced dementia	NS	Qualitative design	Semi-structured, audio-recorded individual interviews	Thematic analysis	Family members' understandings about the terminal nature of dementia
Ashton[4] (2016)	UK	-	-	12	[35-82]	Nursing home	Advanced dementia	Alzheimer's; Huntington's disease; Lewy bodies; Vascular; mixed-type; early onset dementia	Descriptive qualitative design (based on principles of naturalistic inquiry)	Semi-structured, in-depth interviews	Content analysis	Experiences of Advance Care Planning
Beernaert[5] (2016)	NL	6	[67-86]	-	-	Community	NS	Alzheimer's (n=5)	NS	Face-to-face semi-structured interviews	Thematic content and narrative analysis	Palliative care in patients with life-limiting illnesses
Beernaert[6] (2015)	NL	6	[67-86]	-	-	Community	NS	Alzheimer's (n=1)	NS	Face-to-face semi-structured interviews	Thematic content analysis	Family physicians' role in palliative care
Beisecker[7] (1997)	US	-	-	104	63.6 [27-88]	Community; Nursing home; Veterans Administration facility	NS	Alzheimer's (n=16); Mixed-type (n=3); Vascular (n=1)	NS	Semi-structured interviews	NS	Communication between carers and physicians
Black[8] (2009)	US	-	-	34	61 [37-79]	Nursing home	NS	Alzheimer's (n=16); Vascular (n=18); Mixed-type (n=7); Lewy bodies (n=2); Frontotemporal (n=1)	Descriptive qualitative	Face-to-face, Semistructured field guides	Iterative process	SDM's understanding of patient preferences

Study	Country	No. of Person with dementia	Person with dementia age (mean [range])	No. of CG	CG age (mean [range])	Care Setting	Dementia Stage	Dementia Type	Methodology	Qualitative Data Collection	Analysis	Topic
Bonnel[9] (1996)	US	-	-	1	79	Community; Nursing home	Advanced-stage dementia	Alzheimer's (n=20)	Qualitative case report	Broad open-ended interviews	Content analysis	Spouse experience of Alzheimer's Disease
Bosek[10] (2003)	US	-	-	57	NS	Nursing home	NS	Alzheimer's (n=3); Lewy body (n=2); Pick's disease (n=1); Vascular/multi-infarct (n=2); Unspecified (n=6)	NS	Semi structured, in-depth interviews	NS	Having a "good death"
Boyd[11] (2011)	SL	12	NS	12	NS	NS	Early-stage dementia	Alzheimer's 104	NS	Interviews	NS	End-of-Life care
Cahill[12] (2012)	IE	-	-	16	77 [62-88]	Nursing home; hospital	End-stage dementia	Alzheimer's disease	NS	In-depth qualitative interviews	Qualitative thematic analysis	Carer experience at End of Life
Cairns[13] (2012)	UK	-	-	5	[62-84]	Residential care	NS	Alzheimer's late onset (n=4); Mixed-type Alzheimer's (n=4); Alzheimer's of unspecified type (n=1)	Psychodynamic	Semi-structured interviews	NS	Residential care placement
Caron[14] (2005)	CA	-	-	24	[40-83]	Long-term care facility	Advanced-stage dementia	Alzheimer's late onset (n=5); Mixed-type Alzheimer's (n=1)	Grounded theory	Open-ended interviews	Constant-comparative method; line-by-line/dimensional analysis	End-of-Life decision making
Caron[15] (2005)	CA	-	-	24	[40-83]	Long-term care facility	Advanced-stage dementia	NS	Grounded theory	Open-ended interviews	Constant-comparative method; line-by-line/dimensional analysis	End-of-Life decision making
Cronfalk[16] (2017)	SE	-	-	10	NS	Nursing home	NS	NS	Descriptive design	face-to-face interviews	Qualitative content analysis	Nursing home placement
Crowther[17] (2013)	UK	-	-	40	[18-86]	Community; District general hospital; Care home	NS	NS	Grounded Theory and phenomenology.	Unstructured narrative interviewing	NS	End-of-Life care
Davies[18] (2017)	UK	-	-	47	[39-90]	Community; Hospital; Care home; Hospice	NS	NS	Qualitative methods	Face-to-face, in-depth interviews	Thematic analysis	Quality End-of-Life care

Study	Country	No. of Person with dementia	Person with dementia age (mean [range])	No. of CG	CG age (mean [range])	Care Setting	Dementia Stage	Dementia Type	Methodology	Qualitative Data Collection	Analysis	Topic
de Boer[19] (2012)	NL	24	76.3 [65-89]	-	-	Community	Early-stage dementia	NS	NS	Semi-structured interview guides, face-to-face with open-ended questions	Iterative analysis	ACP
de Vries[20] (2013)	UK	-	-	2	NS	Hospital; Hospice	NS	NS	Inductive approach through grounded theory methodology	Single interviews	NS	End-of-Life care
Dening[21] (2012)	UK	-	-	7	NS	NS	NS	NS	NS	Topic guide to lead semi-structured interviews	Thematic content analysis	Barriers to End-of-Life care
Dening[22] (2013)	UK	8	83.3	9	69.2	Community	NS	NS	NS	Modified nominal group technique	Qualitative content analysis	Preferences for End-of-Life care
Dening[23] (2017)	UK	6	77.6 [70-88]	7	73.4 [49-85]	Community	NS	NS	Qualitative approach (naturalistic interpretative)	Semi-structured interview with three open-ended questions	Content analysis	Health-care decision making in dementia
Dickinson[24] (2013)	UK	17	[46-96]	29	[44-89]	NS	Mild to moderate dementia	NS	NS	Semi-structured interviews	Thematic analysis	Views on ACP
Fetherstonh-augh[25] (2017)	AU	-	-	34	57.4	Community; RACF	NS	NS	Qualitative design	Semi-structured face-to-face or telephone interviews	Thematic analysis	Surrogate decision-making
Fleming[26] (2015)	AU	2	NS	11	NS	NS	Severe stage dementia	NS	Mixed-method design	Focus groups	NS	Environmental design at End-of-Life in dementia
Forbes[27] (2012)	CA	5	77.4 [63-95]	14	60.3 [39-91]	Community	NS	NS	Qualitative interpretative descriptive approach	Face-to-face interviews	Lubrosky's thematic analysis	Stages of dementia care journey
Forbes[28] (2000)	US	-	-	28	66 [41-85]	Nursing home	Moderately severe or severe dementia	NS	Naturalistic approach	Qualitative focus groups	Qualitative content analysis	End-of Life decision making

Study	Country	No. of Person with dementia	Person with dementia age (mean [range])	No. of CG	CG age (mean [range])	Care Setting	Dementia Stage	Dementia Type	Methodology	Qualitative Data Collection	Analysis	Topic
Gessert[29] (2006)	US	35	88	38	62	Nursing home	Severe cognitive impairment;	NS	NS	Focus groups with interview guide	NS	Attitudes towards end-of-life decision making
Gessert[30] (2000)	US	-	-	28	NS	Nursing home	Moderately severe to severe dementia	NS	Qualitative design	Focus groups with interview guide	Qualitative content analysis	End-of Life decision making
Givens[31] (2012)	US	-	-	16	62	Nursing home	Advanced dementia	NS	NS	Semi-structured interviews	NS	Carer burden
Glass[32] (2016)	US	-	-	4	[65-80s]	Community; Hospice; Nursing home;	NS	NS	Phenomenological qualitative approach; multiple case design	Narrative interview (interview guide with open-ended questions)	NS	Site for End-of-Life care
Godwin[33] (2009)	UK	12	NS	12	NS	Hopsital wards; Hospice; Nursing home	Advanced dementia	NS	NS	Interviews with open-ended questions	NS	End-of-Life care
Goodman[34] (2013)	UK	18	[68.7-92]	-	-	Care homes	NS	NS	Exploratory qualitative	Semistructured, conversational interviews	NS	Preferences for End-of-Life care
Groen-van de Ven[35] (2016)	NL	23	[62-89]	44	NS	Community; Institutions (sheltered and Nursing homes)	Beginning, moderate, and advanced dementia	NS	Prospective, qualitative, multi-perspective design	Semistructured interviews using an interview guide	Content analysis; timeline method; constant comparative method	Decision trajectories in dementia care
Groen-van de Ven[36] (2016)	NL	23	[62-89]	44	NS	Community; Institutions (sheltered and Nursing homes)	Beginning, moderate, and advanced dementia	NS	Qualitative, multi-perspective design	Semistructured interviews using an interview guide	Content analysis; constant comparative method	Challenges of shared decision making in dementia
Hill[37] (2016)	UK	14	NS	43	NS	NS	Early-stage dementia	NS	Q-methodology	Q-methodology post-sort questions	NS	End-of-Life care
Hirschman[38] (2006)	US	-	-	30	64.3 [42-93]	Community; Nursing home; Assisted living facility	Advanced dementia	NS	NS	Semi-structured interviews	NS	Barriers to substitute decision making
Hirschman[39] (2008)	US	-	-	30	64.3 [42-93]	Community; Nursing home; Assisted living facility	Advanced dementia	NS	NS	Semi-structured interviews	NS	Barriers to /facilitators to advance care planning

Study	Country	No. of Person with dementia	Person with dementia age (mean [range])	No. of CG	CG age (mean [range])	Care Setting	Dementia Stage	Dementia Type	Methodology	Qualitative Data Collection	Analysis	Topic
Gil[40] (2018)	IL	-	-	17	[40-70s]	Hospital	Advanced dementia	NS	NS	Observation and interviews	Thematic approach	End-of-life decision making
Hovland[41] (2016)	US	-	-	30	[45-88]	Community; Hospital; Hospice; Skilled or rehab nursing centre	NS	NS	Qualitative descriptive approach	In-depth, qualitative interviews	Qualitative content analysis	Preparedness for death
Jenning[42] (2017)	SZ	7	71 [73-92]	36	63 [42-85]	Community	Early-stage dementia	NS	NS	Focus groups	NS	Goals for dementia care
Jox[43] (2011)	GER	-	-	32	61 [31-82]	RACF	NS	NS	Qualitative study	Audiotaped verbalisations to vignettes	Qualitative content analysis	Surrogate decision making
Juozapavicius[44] (2001)	US	-	-	20	63.9 [35 - >86]	Community	NS	NS	NS	Interview with guide	Constant comparative method	Caregiving experience in Alzheimer's
Lamahewa[45]	UK	-	-	46	NS	Community; Hospital	NS	NS	Qualitative study	Semi-structured interviews and focus groups	Thematic analysis	Decision-making at end-of-life
Lawrence[46] (2011)	UK	-	-	27	NS	Care home; Community; Continuing care unit; General hospital	NS	NS	NS	In-depth interviews	NS	Dying well and End-of-Life care
Lewis[47] (2014)	US	-	-	11	NS	Community; Hospitals; Hospice; Nursing homes	NS	NS	Descriptive phenomenological method	Interviews	Colaizzi method	Carer's experience with seeking hospice care
Lewis[48] (2015)[a]	US	-	-	101	NS	Community; Hospital; Hospice; Assisted living; Nursing home	NS	NS	Grounded theory	In-person interviews, online interviews, book and blog memoirs of caregivers, and participant observation.	Constant comparative method	Caregiving at End-of-Life; Theory-making
Livingston[49] (2010)	UK	-	-	46	62	Community; Care home	NS	NS	Qualitative study/ Grounded Theory	In-depth, semi-structured interviews	Thematic analysis	Barriers to /facilitators of substitute decision making

Study	Country	No. of Person with dementia	Person with dementia age (mean [range])	No. of CG	CG age (mean [range])	Care Setting	Dementia Stage	Dementia Type	Methodology	Qualitative Data Collection	Analysis	Topic
Livingston[50] (2013)	UK	-	-	20	[33-95]	Care home	NS	NS	NS	Qualitative interview, open questions with an interview guide	Thematic analysis	Intervention study at End-of-Life
Mann[51] (2013)	US	-	-	16	NS	Nursing home	NS	NS	NS	Semi-structured qualitative interviews	Constant comparative method	Do-not-hospitalise orders
Muders[52] (2015)	GER	-	-	85	NS	NS	NS	NS	NS	Cross-sectional survey, open-ended questions	Qualitative content analysis	Carer's experience with caring for Person with dementia at end-of-life
Mulqueen[53] (2017)	IE	NS	NS	-	-	RACF	Mild dementia	NS	NS	Nominal group technique	Manifest content analysis method	Preferences for end-of-life care
Noh[54] (2016)	US	-	-	20	63.4	Community; Other unspecified	NS	NS	NS	Semi-structured telephone or face-to-face interviews	Thematic analysis	Proxies' perception of support in end-of-life decision making
Pasman[55] (2004)	NL	-	-	32	NS	Nursing home	Middle-stage to severe (as per 3 case studies)	NS	NS	Participant observation	Constant comparison method	Decision making on artificial nutrition and hydration
Peacock[56] (2014)	CA	-	-	11	[49-89]	Long-term care home	Advanced dementia	NS	Munhall's methodology for interpretive phenomenology	In-depth, open-ended interviews	Hermeneutic approach	End-of-life dementia caregiving experience
Peacock[57] (2014)	CA	-	-	11	[49-89]	Community; Long-term care home	NS	NS	NS	Open-ended interviews	Qualitative description approach	Dementia caregiving journey of bereaved carers
Poole[58] (2017)	EN	11	NS	25	NS	Community; supported living; group living; standard nursing/residential care	Advanced Dementia	NS	Social constructivist epistemological	Q-sort methodology, with open-ended questions; face-to-face semi-structured qualitative interviews	Thematic analysis	End-of-life care in dementia
Poppe[59] (2013)	UK	12	NS	8	NS	Community	Mild dementia	NS	NS	In-depth, open-ended interviews	Constant comparison method	Advance care planning in early dementia

Study	Country	No. of Person with dementia	Person with dementia age (mean [range])	No. of CG	CG age (mean [range])	Care Setting	Dementia Stage	Dementia Type	Methodology	Qualitative Data Collection	Analysis	Topic
Powers[60] (2008)	US	6	NS	NS	NS	Hospice; Nursing home	NS	NS	Concurrent mixed methods design (Qual strand: ethnographic field study)	Interviews with guides	Content analysis	Palliative end-of-life care in dementia
Robinson[61] (2000)	US	-	-	12	[51-79]	Long-term care facility	Advanced dementia	NS	Phenomenological study	Interviews	VanManen's selective and highlighting approach, with Linguistic transformation?	SDM's experience of implementing and advance care plan
Rosemond[62] (2017)	US	-	-	16	NS	Nursing home	Advanced dementia	NS	Qualitative study	Semi-structured interviews	Qualitative description approach	SDM's experience of goals-of-care decision making
Russell[63] (2008)	AU	-	-	15	[35-75]	Community; Hospital; Nursing home	Advanced to terminal dementia	NS	NS	Semistructured in-depth interviews, with open-ended questions	Thematic analysis	Carer's view on quality of life in advanced-terminal dementia
Saini[64] (2016)	UK	-	-	4	[54-76]	Nursing home	Advanced dementia	NS	Ethnography	Semi-structured interviews using open-ended questions	Thematic analysis	End-of-life discussions
Samia[65] (2012)	US	-	-	26	[<60-84]	Community; Other	NS	NS	Qualitative descriptive study	Focus groups	Content analysis	Learning needs/ challenges of carers participating in psychoeducation program
Sanders[66] (2009)	US	-	-	27	[38-83]	Community; long-term care facility (assisted living or nursing home); hospice	End-stage dementia	NS	Ethnomethodology	Semi-structured interviews	Constant comparative method;	Carers' response to end-stage dementia care
Sarabia-Cobo[67] (2016)	SPN	-	-	84	66 [36-87]	Nursing home	Moderate to severe dementia	NS	Naturalistic approach	Qualitative focus groups (Krueger method)	Qualitative content analysis	End-of-Life decision making
Shanley[68] (2017)	AU	-	-	34	57.4	Community; RACF	NS	NS	Qualitative study	Semi-structured face-to-face or telephone interviews	Thematic analysis	SDM's support needs

Study	Country	No. of Person with dementia	Person with dementia age (mean [range])	No. of CG	CG age (mean [range])	Care Setting	Dementia Stage	Dementia Type	Methodology	Qualitative Data Collection	Analysis	Topic
Shanley[69] (2011)	AU	-	-	15	[35-75]	Community; Hospital; Nursing home	Advanced and terminal stage dementia	NS	NS	Semi-structured in-depth interviews with open-ended questioning	Thematic analysis	Family carer support needs
Shuter[70] (2014)	AU	-	-	13	[54-82]	Community; Hospital; RACF	NS	NS	Kramer's Conceptual Model of Carer Adaptation; Cognitive-based Behavioural Conceptualisation of Complicated Grief	Semi-structured interviews with open-ended questions	Problem-Driven Content Analysis	Grief and health outcomes of dementia carers
Slape[71] (2014)	AU	-	-	10	NS	RACF	NS	NS	Hermeneutic phenomenological approach	Semi-structured in-depth interviews	In-depth qualitative analysis	Spiritual needs of family members
Snyder[72] (2013)[b]	US	-	-	126	59.3	Nursing home	Advanced dementia	NS	NS	Semi-structured interviews, open-ended questions about the advantages and disadvantages	NS	SDM's perceptions of feeding options
Stewart[73] (2011)	US	-	-	2	NS	Residential and nursing care homes	NS	NS	NS	Semi-structured interviews	Framework analysis	ACP
Stewart-Archer[74] (2015)	CA	136	NS	-	-	Community; Supportive housing; Personal care home	NS	NS	NS	Open-ended face-to-face interviews	Qualitative content analysis	End-of-life care preferences
Stirling[75] (2014)	AU	-	-	11	NS	NS	NS	NS	NS	Family interviews	Thematic analysis	Development of "dying" discussion tool
Tarter[76] (2016)	US	-	-	51	62.4	Hospice	NS	NS	NS	Secondary data from audio-recordings of therapy intervention session	Thematic analysis	Hospice pain management
The[77] (2002)	NL	35	[61-98]	32	NS	Nursing home	NS	NS	Ethnography	Comprehensive notes of observations and informal; formal interviews	NS	Withholding artificial administration of fluids and food

Study	Country	No. of Person with dementia	Person with dementia age (mean [range])	No. of CG	CG age (mean [range])	Care Setting	Dementia Stage	Dementia Type	Methodology	Qualitative Data Collection	Analysis	Topic
Thuné-Boyle[78] (2010)	UK	-	-	20	[44-89]	Community; Nursing/ residential home; Hospital	Advanced dementia	NS	Qualitative methodology	Semi-structured interviews	Framework analysis	End-of-life care needs
Treloar[79] (2009)	UK	-	-	14	68.1 [36-91]	Community; Hospital; Nursing care home	Scores of 6-7 (sever to very severe cognitive decline) as rated on Functional Assessment Staging (FAST). Stage 7 is the most advanced category of dementia.	NS	NS	Qualitative semi-structured questionnaire (allowing for free interview)	NS	Palliative and end of life care of dementia at home
van der Steen[80] (2017)	NL	-	-	10	[59-86]	Community; Nursing home	Advanced, terminal phase dementia	NS	NS	Focus groups	Thematic analysis	Palliative care service development for terminal dementia
van Soest-Poortvliet[81] (2015)	NL	-	-	20	61 [46-68]	Nursing home	Score 13 mean (8-20 range) on Bedford Alzheimer Nursing Severity-Scale (BANS-S); (Rating from 7-28; higher score means more severe dementia.	NS	Qualitative descriptive methodology	Explorative, qualitative in-depth interviews	Thematic analysis	ACP
Volicer[82] (2016)	US	-	-	15	NS	Hospice	Advanced dementia	Variant Creutzfeldt-Jakob disease (vCJD)	NS	Focus groups	NS	Acceptability of food-limiting advance directive
Wakunami[83] (2009)	JPN	-	-	4	[59-78]	Community; Hospital; Nursing home	NS		Qualitative methodology	Semi-structured interviews	NS	Introducing end-of-life care to families

Study	Country	No. of Person with dementia	Person with dementia age (mean [range])	No. of CG	CG age (mean [range])	Care Setting	Dementia Stage	Dementia Type	Methodology	Qualitative Data Collection	Analysis	Topic
Wladkowski[84] (2016)[c]	US	-	-	24	63 [48–78]	Community; Assisted living facility; Skilled nursing facility; Hospice home	NS	NS	NS	Semi-structured face-to-face or telephone interviews	Thematic analysis	Carer grief over live hospice discharge

Abbreviations and definitions: ACP, advance care planning; AU, Australia; CA, Canada, Colaizzi method, analytical method using descriptive phenomenology; content analysis, deductive methodology that involves identification of codes prior to searching for their occurrence in the data; EoL, end of life; ethnography, to discover and describe individual social and cultural groups; GER, Germany; IE, Ireland; IS, Israel; JPN, Japan; mixed methods, include quantitative and qualitative methods; NL, Netherlands; nominal group technique, semi-evaluation method involving qualitative and quantitative approaches; NS, not state; phenomenology, to study peoples' understanding and interpretations of their experiences in their own terms and emphasizing these as explanations for their actions; Q-methodology, a mixed method combining qualitative and quantitative techniques to study subjectivity; RACF, residential aged care facility; SDM, substitute decision maker; SE, Sweden; SPN, Spain; SWZ, Switzerland; thematic analysis, concepts and theories are inductively derived from the data; UK, United Kingdom; US, United States.

a theoretical sample of 101 participants

b demographics based on 127 participants

c only 18 of 24 participants reported their ages

References

1. Albinsson L, Strang P. Existential concerns of families of late-stage dementia patients: questions of freedom, choices, isolation, death, and meaning. Journal Of Palliative Medicine. 2003;6(2):225-35.
2. Almberg BE, Grafström M, Winblad B. Caregivers of relatives with dementia: experiences encompassing social support and bereavement. Aging & Mental Health. 2000;4(1):82-9.
3. Andrews S, McInerney F, Toye C, Parkinson C-A, Robinson A. Knowledge of Dementia: Do family members understand dementia as a terminal condition? Dementia. 2017;16(5):556-75.
4. Ashton SE, Roe B, Jack B, McClelland B. End of life care: The experiences of advance care planning amongst family caregivers of people with advanced dementia–A qualitative study. Dementia. 2016;15(5):958-75.
5. Beernaert K, Deliens L, De Vleminck A, Devroey D, Pardon K, Van den Block L, et al. Is there a need for early palliative care in patients with life-limiting illnesses? Interview study with patients about experienced care needs from diagnosis onward. American Journal of Hospice & Palliative Medicine. 2016;33(5):489-97.
6. Beernaert K, Van den Block L, Van Thienen K, Devroey D, Pardon K, Deliens L, et al. Family physicians' role in palliative care throughout the care continuum: stakeholder perspectives. Family Practice. 2015;32(6):694-700.
7. Beisecker AE, Chrisman SK, Wright LJ. Perceptions of family caregivers of persons with Alzheimer's disease: communication with physicians. American Journal of Alzheimer's Disease. 1997;12(2):73-83.
8. Black BS, Fogarty LA, Phillips H, Finucane T, Loreck DJ, Baker A, et al. Surrogate decision makers' understanding of dementia patients' prior wishes for end-of-life care. Journal Of Aging And Health. 2009;21(4):627-50.
9. Bonnel WB. Not gone and not forgotten: a spouse's experience of late-stage Alzheimer's disease. Journal of Psychosocial Nursing & Mental Health Services. 1996;34(8):23-40.
10. Bosek MSD, Lowry E, Lindeman DA, Burck R, Gwyther LP. Promoting a good death for persons with dementia in nursing facilities: family caregivers' perspectives. JONA's Healthcare Law, Ethics & Regulation. 2003;5(2):34-41.
11. Boyd R. End-of-life care. Discussing end-of-life care with people with dementia: a word of caution. Mental Health Nursing. 2011;31(1):14-7.
12. Cahill S, Doran D, Watson M. Guidelines for nursing homes delivering end-of-life care to residents with dementia across the island of Ireland. Quality in Ageing & Older Adults. 2012;13(1):60-70.
13. Cairns M. In sickness and in health: An exploration of some of the unconscious processes involved in the decision by family caregivers to place a family member with dementia in residential care. Psychoanalytic Psychotherapy. 2012;26(1):34-47.
14. Caron CD, Griffith J, Arcand M. Decision making at the end of life in dementia: How family caregivers perceive their interactions with health care providers in long-term-care settings. Journal of Applied Gerontology. 2005;24(3):231-47.
15. Caron CD, Griffith J, Arcand M. End-of-life decision making in dementia: The perspective of family caregivers. Dementia: The International Journal of Social Research and Practice. 2005;4(1):113-36.
16. Cronfalk BS, Ternestedt B-M, Norberg A. Being a close family member of a person with dementia living in a nursing home. Journal Of Clinical Nursing. 2017.

17. Crowther J, Wilson KCM, Horton S, Lloyd-Williams M. Compassion in healthcare - lessons from a qualitative study of the end of life care of people with dementia. Journal Of The Royal Society Of Medicine. 2013;106(12):492-7.
18. Davies N, Rait G, Maio L, Iliffe S. Family caregivers' conceptualisation of quality end-of-life care for people with dementia: A qualitative study. Palliat Med. 2016.
19. de Boer ME, Dröes R-M, Jonker C, Eefsting JA, Hertogh CM. Thoughts on the future: The perspectives of elderly people with early-stage Alzheimer's disease and the implications for advance care planning. AJOB Primary Research. 2012;3(1):14-22.
20. de Vries K, Sque M, Bryan K, Abu-Saad H. Variant Creutzfeldt-Jakob disease: need for mental health and palliative care team collaboration. International Journal Of Palliative Nursing. 2003;9(12):512-20.
21. Dening KH, Greenish W, Jones L, Mandal U, Sampson EL. Barriers to providing end-of-life care for people with dementia: a whole-system qualitative study. BMJ Supportive & Palliative Care. 2012;2(2):103-7.
22. Dening KH, Jones L, Sampson EL. Preferences for end-of-life care: a nominal group study of people with dementia and their family carers. Palliative medicine. 2013;27(5):409-17.
23. Dening KH, King M, Jones L, Sampson EL. Healthcare decision-making: past present and future, in light of a diagnosis of dementia. International Journal of Palliative Nursing. 2017;23(1):4-11.
24. Dickinson C, Bamford C, Exley C, Emmett C, Hughes J, Robinson L. Planning for tomorrow whilst living for today: the views of people with dementia and their families on advance care planning. International psychogeriatrics. 2013;25(12):2011-21.
25. Fetherstonhaugh D, McAuliffe L, Bauer M, Shanley C. Decision-making on behalf of people living with dementia: How do surrogate decision-makers decide? Journal of Medical Ethics: Journal of the Institute of Medical Ethics. 2017;43(1):35-40.
26. Fleming R, Kelly F, Stillfried G. 'I want to feel at home': Establishing what aspects of environmental design are important to people with dementia nearing the end of life Palliative care in other conditions. BMC Palliative Care. 2015;14(1).
27. Forbes DA, Finkelstein S, Blake CM, Gibson M, Morgan DG, Markle-Reid M, et al. Knowledge exchange throughout the dementia care journey by Canadian rural community-based health care practitioners, persons with dementia, and their care partners: an interpretive descriptive study. Rural And Remote Health. 2012;12(4):2201-.
28. Forbes S, Bern-Klug M, Gessert C. End-of-Life Decision Making for Nursing Home Residents with Dementia. Journal of Nursing Scholarship. 2000;32(3):251-8.
29. Gessert CE, Elliott BA, Peden-McAlpine C. Family Decision-Making for Nursing Home Residents With Dementia: Rural-Urban Differences. The Journal of Rural Health. 2006;22(1):1-8.
30. Gessert CE, Forbes S, Bern-Klug M. Planning end-of-life care for patients with dementia: roles of families and health professionals. Omega. 2000;42(4):273-91.
31. Givens JL, Lopez RP, Mazor KM, Mitchell SL, Givens JL, Lopez RP, et al. Sources of stress for family members of nursing home residents with advanced dementia. Alzheimer Disease & Associated Disorders. 2012;26(3):254-9.
32. Glass AP. Family Caregiving and the Site of Care: Four Narratives About End-of-Life Care for Individuals with Dementia. Journal Of Social Work In End-Of-Life & Palliative Care. 2016;12(1-2):23-46.
33. Godwin B, Waters H. ‘In solitary confinement’: Planning end-of-life well-being with people with advanced dementia, their family and professional carers. Mortality. 2009;14(3):265-85.

34. Goodman C, Amador S, Elmore N, Machen I, Mathie E. Preferences and priorities for ongoing and end-of-life care: a qualitative study of older people with dementia resident in care homes. International Journal Of Nursing Studies. 2013;50(12):1639-47.
35. Groen-van de Ven L, Smits C, Oldewarris K, Span M, Jukema J, Eefsting J, et al. Decision Trajectories in Dementia Care Networks: Decisions and Related Key Events. Research On Aging. 2016.
36. Groen-van de Ven L, Smits C, Span M, Jukema J, Coppoolse K, de Lange J, et al. The challenges of shared decision making in dementia care networks. International Psychogeriatrics. 2016:1-15.
37. Hill SR, Mason H, Poole M, Vale L, Robinson L. What is important at the end of life for people with dementia? The views of people with dementia and their carers. International journal of geriatric psychiatry. 2016.
38. Hirschman KB, Kapo JM, Karlawish JH. Why doesn't a family member of a person with advanced dementia use a substituted judgment when making a decision for that person? The American journal of geriatric psychiatry. 2006;14(8):659-67.
39. Hirschman KB, Kapo JM, Karlawish JH, Hirschman KB, Kapo JM, Karlawish JHT. Identifying the factors that facilitate or hinder advance planning by persons with dementia. Alzheimer Disease & Associated Disorders. 2008;22(3):293-8.
40. Gil E, Agmon M, Hirsch A, Ziv M, Zisberg A. Dilemmas for guardians of advanced dementia patients regarding tube feeding. Age and ageing. 2018;47(1):138-43.
41. Hovland-Scafe CA, Kramer BJ. Preparedness for Death: How Caregivers of Elders With Dementia Define and Perceive its Value. The Gerontologist. 2016.
42. Jennings LA, Palimaru A, Corona MG, Cagigas XE, Ramirez KD, Zhao T, et al. Patient and caregiver goals for dementia care. Quality Of Life Research: An International Journal Of Quality Of Life Aspects Of Treatment, Care And Rehabilitation. 2017;26(3):685-93.
43. Jox RJ, Denke E, Hamann J, Mendel R, Forstl H, Borasio GD. Surrogate decision making for patients with end-stage dementia. International journal of geriatric psychiatry. 2012;27(10):1045-52.
44. Juozapavicius KP, Weber JA. A reflective study of Alzheimer's caregivers. American Journal Of Alzheimer's Disease And Other Dementias. 2001;16(1):11-20.
45. Lamahewa K, Mathew R, Iliffe S, Wilcock J, Manthorpe J, Sampson EL, et al. A qualitative study exploring the difficulties influencing decision making at the end of life for people with dementia. Health Expectations. 2018;21(1):118-27.
46. Lawrence V, Samsi K, Murray J, Harari D, Banerjee S. Dying well with dementia: qualitative examination of end-of-life care. The British Journal Of Psychiatry: The Journal Of Mental Science. 2011;199(5):417-22.
47. Lewis LF. Caregivers' experiences seeking hospice care for loved ones with dementia. Qualitative Health Research. 2014;24(9):1221-31.
48. Lewis LF. Caregiving for a Loved One With Dementia at the End of Life. American Journal of Alzheimer's Disease & Other Dementias. 2015;30(5):488-96.
49. Livingston G, Leavey G, Manela M, Livingston D, Rait G, Sampson E, et al. Making decisions for people with dementia who lack capacity: qualitative study of family carers in UK. BMJ (Clinical Research Ed). 2010;341:c4184-c.
50. Livingston G, Lewis-Holmes E, Pitfield C, Manela M, Chan D, Constant E, et al. Improving the end-of-life for people with dementia living in a care home: an intervention study. International Psychogeriatrics. 2013;25(11):1849-58.
51. Mann E, Goff SL, Colon-Cartagena W, Bellantonio S, Rothberg MB. Do-not-hospitalize orders for individuals with advanced dementia: healthcare proxies' perspectives. Journal Of The American Geriatrics Society. 2013;61(9):1568-73.

52. Muders P, Zahrt-Omar CA, Bussmann S, Haberstroh J, Weber M. Support for families of patients dying with dementia: a qualitative analysis of bereaved family members' experiences and suggestions. Palliative & Supportive Care. 2015;13(3):435-42.
53. Mulqueen K, Coffey A. Preferences of residents with dementia for end of life care. Nursing Older People. 2017;29(2):26-30.
54. Noh H, Kwak J. End-of-life decision making for persons with dementia: Proxies' perception of support. Dementia (London, England). 2016.
55. Pasman HRW, The BAM, Onwuteaka-Philipsen BD, Ribbe MW, van der Wal G. Participants in the decision making on artificial nutrition and hydration to demented nursing home patients: A qualitative study. Journal of Aging Studies. 2004;18(3):321-35.
56. Peacock S, Duggleby W, Koop P. The lived experience of family caregivers who provided end-of-life care to persons with advanced dementia. Palliative & Supportive Care. 2014;12(2):117-26.
57. Peacock SC, Hammond-Collins K, Forbes DA. The journey with dementia from the perspective of bereaved family caregivers: a qualitative descriptive study. BMC Nursing. 2014;13(1):42-.
58. Poole M, Bamford C, McLellan E, Lee RP, Exley C, Hughes JC, et al. End-of-life care: A qualitative study comparing the views of people with dementia and family carers. Palliat Med. 2017;0(0):12.
59. Poppe M, Burleigh S, Banerjee S. Qualitative evaluation of advanced care planning in early dementia (ACP-ED). Plos One. 2013;8(4):e60412-e.
60. Powers BA, Watson NM. Meaning and practice of palliative care for nursing home residents with dementia at end of life. American Journal Of Alzheimer's Disease And Other Dementias. 2008;23(4):319-25.
61. Robinson EM. Wives' struggle in living through treatment decisions for husbands with advanced Alzheimer's disease. Journal Of Nursing Law. 2000;7(1):21-39.
62. Rosemond C, Hanson LC, Zimmerman S. Goals of Care or Goals of Trust? How Family Members Perceive Goals for Dying Nursing Home Residents. Journal Of Palliative Medicine. 2017;20(4):360-5.
63. Russell C, Middleton H, Shanley C. Dying with dementia: the views of family caregivers about quality of life. Australasian Journal On Ageing. 2008;27(2):89-92.
64. Saini G, Sampson EL, Davis S, Kupeli N, Harrington J, Leavey G, et al. An ethnographic study of strategies to support discussions with family members on end-of-life care for people with advanced dementia in nursing homes. BMC Palliative Care. 2016;15:55-.
65. Samia LW, Hepburn K, Nichols L. "Flying by the seat of our pants": what dementia family caregivers want in an advanced caregiver training program. Research In Nursing & Health. 2012;35(6):598-609.
66. Sanders S, Butcher HK, Swails P, Power J. Portraits of caregivers of end-stage dementia patients receiving hospice care. Death Studies. 2009;33(6):521-56.
67. Sarabia-Cobo CM, Pérez V, de Lorena P, Nuñez MJ, Domínguez E. Decisions at the end of life made by relatives of institutionalized patients with dementia. Applied Nursing Research: ANR. 2016;31:e6-e10.
68. Shanley C, Fetherstonhaugh D, McAuliffe L, Bauer M, Beattie E. Providing support to surrogate decision-makers for people living with dementia: Healthcare professional, organisational and community responsibilities. Health & Social Care In The Community. 2017.
69. Shanley C, Russell C, Middleton H, Simpson-Young V. Living through end-stage dementia: The experiences and expressed needs of family carers. Dementia (14713012). 2011;10(3):325-40.

70. Shuter P, Beattie E, Edwards H. An exploratory study of grief and health-related quality of life for caregivers of people with dementia. American Journal of Alzheimer's Disease and other Dementias. 2014;29(4):379-85.
71. Slape J. Dementia and palliative care: The spiritual needs of family members. Journal of Religion, Spirituality & Aging. 2014;26(2-3):215-30.
72. Snyder EA, Caprio AJ, Wessell K, Lin FC, Hanson LC. Impact of a decision aid on surrogate decision-makers' perceptions of feeding options for patients with dementia. Journal Of The American Medical Directors Association. 2013;14(2):114-8.
73. Stewart F, Goddard C, Schiff R, Hall S. Advanced care planning in care homes for older people: a qualitative study of the views of care staff and families. Age And Ageing. 2011;40(3):330-5.
74. Stewart-Archer LA, Afghani A, Toye CM, Gomez FA. Dialogue on Ideal End-of-Life Care for Those With Dementia. The American Journal Of Hospice & Palliative Care. 2015;32(6):620-30.
75. Stirling C, McLnerney F, Andrews S, Ashby M, Toye C, Donohue C, et al. A tool to aid talking about dementia and dying--development and evaluation. Collegian (Royal College Of Nursing, Australia). 2014;21(4):337-43.
76. Tarter R, Demiris G, Pike K, Washington K, Parker Oliver D. Pain in Hospice Patients With Dementia: The Informal Caregiver Experience. American Journal Of Alzheimer's Disease And Other Dementias. 2016;31(6):524-9.
77. The AM, Pasman R, Onwuteaka-Philipsen B, Ribbe M, Van der Wal G. Withholding the artificial administration of fluids and food from elderly patients with dementia: Ethnographic study. British Medical Journal. 2002;325(7376):1326-9.
78. Thuné-Boyle IC, Sampson EL, Jones L, King M, Lee DR, Blanchard MR. Challenges to improving end of life care of people with advanced dementia in the UK. Dementia. 2010;9(2):259-84.
79. Treloar A, Crugel M, Adamis D. Palliative and end of life care of dementia at home is feasible and rewarding: results from the 'Hope for Home' study. Dementia (14713012). 2009;8(3):335-47.
80. Van der Steen JT, Lemos Dekker N, Gijsberts MJHE, Vermeulen LH, Mahler MM, The BAM. Palliative care for people with dementia in the terminal phase: a mixed-methods qualitative study to inform service development. BMC Palliat Care [Internet]. 2017 [cited 2018 Jan 16];16 (1)(28).
81. van Soest-Poortvliet MC, van der Steen JT, Gutschow G, Deliens L, Onwuteaka-Philipsen BD, de Vet HCW, et al. Advance Care Planning in Nursing Home Patients With Dementia: A Qualitative Interview Study Among Family and Professional Caregivers. Journal Of The American Medical Directors Association. 2015;16(11):979-89.
82. Volicer L, Stets K. Acceptability of an Advance Directive That Limits Food and Liquids in Advanced Dementia. The American journal of hospice & palliative care. 2016;33(1):55-63.
83. Wakunami M, Kawabata H, Murakami M, Maezawa M. Families' acceptance of near death: a qualitative study of the process for introducing end-of-life care. Geriatrics & Gerontology International. 2009;9(2):140-7.
84. Wladkowski SP. Live Discharge from Hospice and the Grief Experience of Dementia Caregivers. Journal of Social Work in End-of-Life & Palliative Care. 2016;12(1/2):47-62.
